# Racial and Ethnic Disparities in Cancer Care During the COVID-19 Pandemic

**DOI:** 10.1001/jamanetworkopen.2022.22009

**Published:** 2022-07-14

**Authors:** Manali I. Patel, Jacqueline M. Ferguson, Eida Castro, Cristina D. Pereira-Estremera, Guillermo N. Armaiz-Peña, Ysabel Duron, Fay Hlubocky, Analynn Infantado, Bles Nuqui, Donna Julian, Nii Nortey, Alexandra Steck, Melissa Bondy, Shail Maingi

**Affiliations:** 1 Division of Oncology, Department of Medicine, Stanford University, Stanford, California; 2 Medical Services, Veterans Affairs Palo Alto Health Care System, Palo Alto, California; 3 Department of Epidemiology and Population Health, Stanford University, Stanford, California; 4 Department of Psychology, Ponce Health Sciences University, Ponce Research Institute, Ponce, Puerto Rico; 5 Battle Creek Veterans Affairs Medical Center–Clinical Neuropsychology Clinic, Battle Creek, Michigan; 6 The Latino Cancer Institute, San Jose, California; 7 Department of Medicine, University of Chicago, Chicago, Illinois; 8 St Peter’s Health Partners Cancer Care, Albany, New York; 9 Dana Farber Cancer Institute, Boston, Massachusetts

## Abstract

**Importance:**

The full effect of the COVID-19 pandemic on cancer care disparities, particularly by race and ethnicity, remains unknown.

**Objectives:**

To assess whether the race and ethnicity of patients with cancer was associated with disparities in cancer treatment delays, adverse social and economic effects, and concerns during the COVID-19 pandemic and to evaluate trusted sources of COVID-19 information by race and ethnicity.

**Design, Setting, and Participants:**

This national survey study of US adults with cancer compared treatment delays, adverse social and economic effects, concerns, and trusted sources of COVID-19 information by race and ethnicity from September 1, 2020, to January 12, 2021.

**Exposures:**

The COVID-19 pandemic.

**Main Outcomes and Measures:**

The primary outcome was delay in cancer treatment by race and ethnicity. Secondary outcomes were duration of delay, adverse social and economic effects, concerns, and trusted sources of COVID-19 information.

**Results:**

Of 1639 invited respondents, 1240 participated (75.7% response rate) from 50 US states, the District of Columbia, and 5 US territories (744 female respondents [60.0%]; median age, 60 years [range, 24-92 years]; 266 African American or Black [hereafter referred to as *Black*] respondents [21.5%]; 186 Asian respondents [15.0%]; 232 Hispanic or Latinx [hereafter referred to as *Latinx*] respondents [18.7%]; 29 American Indian or Alaska Native, Native Hawaiian, or multiple races [hereafter referred to as *other*] respondents [2.3%]; and 527 White respondents [42.5%]). Compared with White respondents, Black respondents (odds ratio [OR], 6.13 [95% CI, 3.50-10.74]) and Latinx respondents (OR, 2.77 [95% CI, 1.49-5.14]) had greater odds of involuntary treatment delays, and Black respondents had greater odds of treatment delays greater than 4 weeks (OR, 3.13 [95% CI, 1.11-8.81]). Compared with White respondents, Black respondents (OR, 4.32 [95% CI, 2.65-7.04]) and Latinx respondents (OR, 6.13 [95% CI, 3.57-10.53]) had greater odds of food insecurity and concerns regarding food security (Black respondents: OR, 2.02 [95% CI, 1.34-3.04]; Latinx respondents: OR, 2.94 [95% CI, [1.86-4.66]), financial stability (Black respondents: OR, 3.56 [95% CI, 1.79-7.08]; Latinx respondents: OR, 4.29 [95% CI, 1.98-9.29]), and affordability of cancer treatment (Black respondents: OR, 4.27 [95% CI, 2.20-8.28]; Latinx respondents: OR, 2.81 [95% CI, 1.48-5.36]). Trusted sources of COVID-19 information varied significantly by race and ethnicity.

**Conclusions and Relevance:**

In this survey of US adults with cancer, the COVID-19 pandemic was associated with treatment delay disparities and adverse social and economic effects among Black and Latinx adults. Partnering with trusted sources may be an opportunity to overcome such disparities.

## Key Points

**Question:**

Did racial and ethnic minority adults with cancer in the United States experience more cancer care delays and adverse social and economic effects than White adults during the COVID-19 pandemic?

**Findings:**

In this survey study of 1240 US adults with cancer, Black and Latinx adults reported experiencing higher rates of delayed cancer care and more adverse social and economic effects than White adults.

**Meaning:**

This study suggests that the COVID-19 pandemic is associated with disparities in the receipt of timely cancer care among Black and Latinx adults.

## Teaser

This survey study assesses whether the race and ethnicity of patients with cancer was associated with disparities in cancer treatment delays and adverse social and economic effects during the COVID-19 pandemic.

## Introduction

The COVID-19 pandemic delayed cancer screening^1,2^ and surgical procedures^3^ worldwide, with unknown implications for cancer mortality rates. Before the COVID-19 pandemic, race and ethnicity–based disparities were prevalent in the United States, with lower rates of timely, evidence-based cancer care^4,5,6^ and higher premature death rates among Black and Latinx adults than other racial and ethnic groups.^7,8^ The COVID-19 pandemic highlighted the association of systemic racism and associated inequities in the structural, economic, and socioenvironmental system with these long-standing health disparities.^9,10^ However, the full effect of the COVID-19 pandemic on disparities in cancer care and cancer deaths remains unknown.^11,12^

Since March 2020, Black and Latinx adults have experienced the highest rates of excess morbidity and mortality from COVID-19^13^ and other causes^14^ as well as unemployment.^15^ Medical care disruptions, including cancer screening and treatment delays among Black and Latinx adults with cancer,^16,17^ and disparate adverse social and economic effects fuel concerns regarding the presence of more advanced cancer stages at diagnosis, avoidable cancer deaths,^18,19,20^ and widening of existing race and ethnicity–based disparities in cancer.

To our knowledge, this is the first national study evaluating the association of the COVID-19 pandemic with patient-reported cancer care delays, concerns, and adverse social economic effects by race and ethnicity. Specifically, we sought to evaluate whether racial and ethnic minority adults with cancer, compared with White adults with cancer, experienced more cancer care disruptions, concerns regarding health outcomes, adverse social and economic effects, and concerns regarding the association of COVID-19 with adverse social and economic factors. We also sought to evaluate trusted sources of COVID-19 information by race and ethnicity.

## Methods

We conducted a cross-sectional survey study using a 74-question online survey in English, Spanish, Vietnamese, Chinese, and Hindi to assess patient-reported experiences from September 1, 2020, to January 12, 2021. The survey collected the following: (1) demographic characteristics (eg, gender identity, race and ethnicity, age, income, educational level, and insurance status), (2) clinical characteristics (eg, cancer diagnosis, stage, and treatment), (3) modifications in care, (4) adverse social and economic effects, (5) concerns regarding cancer and other health outcomes, (6) concerns regarding future adverse social and economic effects, and (7) trusted sources of COVID-19 information (eAppendix in the Supplement). Individuals 18 years of age or older with cancer who consented to the study procedures were invited to participate and received no compensation for their participation. Participants were provided with a consent form online and if they participated in the survey, they had consented to participation. Date of diagnosis was not a consideration for inclusion. All survey responses were anonymous unless participants voluntarily provided their name and contact information. The Stanford University School of Medicine institutional review board reviewed and approved the study. This study followed the American Association for Public Opinion Research (AAPOR) reporting guideline for survey studies.^21^

We used a variety of techniques to distribute the survey, including a virtual snowball sampling technique. We distributed the survey link directly to participants in collaboration with clinicians and through online listservs in collaboration with patient advocacy groups and organizations, including the American Society of Clinical Oncology, the Latino Cancer Institute, the Susan G. Komen Foundation, and the International Association for the Study of Lung Cancer, with an emphasis on recruitment of underrepresented racial and ethnic groups. We also promoted the survey on social media. The response rate was calculated among those who directly received the survey link.

We used multivariable logistic regression to estimate odds ratios (ORs) and 95% CIs for 21 factors associated with delays in cancer care, concerns regarding health outcomes, experiences and concerns regarding adverse social and economic effects, and trusted sources of COVID-19 information. We combined self-reported race and ethnicity to create categories of African American or Black (hereafter referred to as *Black*), Asian, Hispanic or Latinx (hereafter referred to as *Latinx*), other (comprised of American Indian or Alaska Native, Native Hawaiian, or multiple races), and White non-Latinx (hereafter referred to as *White*). We calculated all ORs relative to White participants. We adjusted models for a priori selected sociodemographic variables (gender identity, age, educational level, income, insurance status, and place of residence) and clinical variables (cancer diagnosis and stage) associated with delays in care.^22,23^

To account for respondents with unknown cancer stage, we imputed unknown values for cancer stage using multiple imputation^24^ over 100 simulated iterations based on the age, gender identity, place of residence, income, educational level, insurance, and cancer diagnosis of each respondent. In 2 sensitivity analyses, we coded unknown cancer stage as a separate category within the cancer stage variable and conducted a complete-case analysis among participants with a reported known cancer stage. All analyses were 2-sided and conducted using Stata, version 16 (StataCorp LLC), with *P* < .05 considered statistically significant.

## Results

We distributed survey links directly to 1639 individuals; 1240 participated (75.7% response rate), 1128 [91.0%] completed the full survey, and 112 [9.0%] completed 87% of the survey (eFigure in the Supplement). We included 1240 respondents in the analyses.

### Demographic Characteristics

Table 1 lists the demographic and clinical characteristics of the 1240 study participants (186 Asian participants [15.0%]; 266 Black participants [21.5%]; 232 Latinx participants [18.7%]; 29 participants of other race or ethnicity [2.3%], which comprised 3 Native Hawaiian participants [0.2%], 2 Alaska Native participants [0.2%], 9 American Indian participants [0.7%], and 15 participants of multiple races [1.2%]; and 527 White participants [42.5%]; 467 male participants [37.7%], 744 female participants [60.0%], and 29 nonbinary participants [2.3%]; median age, 60 years [range 24-92 years]). Respondents represented 50 US states, the District of Columbia, and 5 US territories, including American Samoa, Guam, Northern Mariana Islands, Puerto Rico, and the US Virgin Islands. A higher proportion of Asian respondents (80 of 186 [43.0%]), Latinx respondents (86 of 232 [37.1%]), and respondents of other race or ethnicity (15 [51.7%]) resided in the West, and a higher proportion of Black respondents (93 [35.0%]) resided in the South.

**Table 1.** Demographic and Clinical Characteristics of the Study Participants by Race and Ethnicity in a National Survey Study of 1240 Adults With Cancer in the US From September 1, 2020, to January 12, 2021

Characteristic	Participants, No. (%)										
	Total (N = 1240)	African American or Black (n = 266 [21.5%])		Asian (n = 186 [15.0%])		Hispanic or Latinx (n = 232 [18.7%])		White (n = 527 [42.5%])		Other (n = 29 [2.3%])^a^	
Gender											
Male	467 (37.7)	153 (57.5)		104 (55.9)		76 (32.8)		118 (22.4)		16 (55.2)	
Female	744 (60.0)	111 (41.7)		80 (43.0)		148 (63.8)		394 (74.8)		11 (37.9)	
Nonbinary	29 (2.3)	2 (0.8)		2 (1.1)		8 (3.4)		15 (2.8		2 (6.9)	
Age, median (range), y	60 (24-92)	59 (31-88)		62 (29-72)		60 (29-92)		65 (24-88)		59 (36-86)	
Place of residence											
Northeast	247 (19.9)	49 (18.4)		37 (19.9)		44 (18.4)		115 (21.8)		2 (6.9)	
Midwest	236 (19.0)	54 (20.3)		28 (15.1)		26 (11.2)		125 (23.7)		3 (10.3)	
South	322 (26.0)	93 (35.0)		35 (18.8)		56 (24.1)		132 (25.0)		6 (20.7)	
West	386 (31.1)	62 (23.3)		80 (43.0)		86 (37.1)		143 (27.1)		15 (51.8)	
Territory (Puerto Rico, Guam, American Samoa, Northern Mariana Islands, and US Virgin Islands)	49 (4.0)	8 (3.0)		6 (3.2)		20 (8.6)		12 (2.3)		3 (10.3)	
Marital status											
Married	672 (54.2)	161 (60.5)		99 (53.2)		93 (40.1)		304 (57.7)		15 (51.7)	
Never married	86 (6.9)	10 (3.8)		8 (4.3)		12 (5.2)		55 (10.4)		1 (3.4)	
Separated	54 (4.4)	14 (5.3)		5 (2.7)		13 (5.6)		18 (3.4)		4 (13.8)	
Divorced	290 (23.4)	59 (22.2)		63 (33.9)		81 (34.9)		80 (15.2)		7 (24.1)	
Widowed	32 (2.6)	5 (1.9)		4 (2.2)		6 (2.6)		17 (3.2)		0	
Unmarried couple	48 (3.9)	6 (2.3)		2 (1.1)		9 (3.9)		29 (5.5)		2 (6.9)	
Prefer not to answer	58 (4.7)	11 (4.1)		5 (2.7)		18 (7.8)		24 (4.6)		0	
No. in household											
1 (Lives alone)	94 (7.6)	11 (4.1)		4 (2.2)		11 (4.7)		64 (12.1)		4 (13.8)	
2	671 (54.1)	156 (58.7)		99 (53.2)		98 (42.2)		302 (57.3)		16 (55.2)	
3	374 (30.2)	80 (30.1)		77 (41.4)		107 (46.1)		102 (19.4)		8 (27.6)	
≥4	101 (8.2)	19 (7.1)		6 (3.2)		16 (6.9)		59 (11.2)		1 (3.5)	
Household with a member ≤18 y	816 (65.8)	230 (86.5)		166 (89.2)		188 (81.0)		213 (40.4)		19 (65.5)	
Household with a member ≥65 y	860 (69.4)	231 (86.8)		170 (91.4)		175 (75.4)		264 (50.1)		20 (69.0)	
Annual household income, $											
<25 000	122 (9.8)	39 (14.7)		18 (9.7)		18 (7.8)		44 (8.4)		3 (10.3)	
25 000-34 999	105 (8.5)	36 (13.5)		5 (2.7)		7 (3.0)		54 (10.3)		3 (10.3)	
35 000-49 999	167 (13.5)	25 (9.4)		42 (22.6)		43 (18.5)		52 (9.9)		5 (17.2)	
50 000-99 999	616 (49.7)	119 (44.7)		106 (57.0)		139 (60.0)		243 (46.1)		9 (31.0)	
≥100 000	162 (13.1)	10 (3.8)		10 (5.4)		25 (10.8)		113 (21.4)		4 (13.8)	
Prefer not to answer	68 (5.5)	37 (13.9)		5 (2.7)		0		21 (4.0)		5 (17.2)	
Educational level											
<High school	303 (24.4)	69 (25.9)		16 (8.6)		134 (57.8)		74 (14.0)		10 (34.5)	
High school degree	149 (12.0)	41 (15.4)		37 (19.9)		33 (14.2)		38 (7.2)		0	
Some college	85 (6.9)	24 (9.0)		2 (1.1)		4 (1.7)		52 (9.9)		3 (10.3)	
Bachelor’s degree	354 (28.6)	66 (24.8)		108 (58.1)		26 (11.2)		152 (28.8)		2 (6.9)	
Master’s degree	190 (15.3)	25 (9.4)		14 (7.5)		29 (12.5)		117 (22.2)		5 (17.2)	
Doctorate degree	91 (7.3)	4 (1.5)		4 (2.2)		6 (2.6)		73 (13.9)		4 (13.8)	
Prefer not to answer	68 (5.5)	37 (13.9)		5 (2.7)		0		21 (3.8)		5 (17.2)	
Employment											
Full-time	571 (46.0)	122 (45.9)		110 (59.1)		184 (79.3)		146 (27.7)		9 (31.0)	
Part-time	112 (9.0)	13 (4.9)		10 (5.4)		2 (0.9)		86 (16.3)		1 (3.5)	
Disabled	94 (7.6)	7 (2.6)		4 (2.2)		16 (6.9)		61 (11.6)		6 (20.7)	
Retired	196 (15.8)	18 (6.8)		10 (5.4)		24 (10.3)		138 (26.2)		6 (20.7)	
Unemployed	198 (16.0)	106 (39.8)		52 (28.0)		6 (2.6)		27 (5.1)		7 (24.1)	
Not looking for work	152 (76.8)	104 (98.1)		16 (30.7)		5 (83.3)		21 (77.8)		6 (85.7)	
Looking for work	46 (23.2)	2 (1.9)		36 (69.2)		1 (16.7)		6 (22.2)		1 (14.3)	
Prefer not to answer	69 (5.6)	0		0		0		69 (13.1)		0	
Insurance											
Medicaid	315 (25.4)	46 (17.3)		66 (35.5)		153 (66.0)		43 (8.2)		7 (24.1)	
Medicare	342 (27.6)	105 (39.5)		14 (7.5)		10 (4.3)		211 (40.0)		2 (6.9)	
Private or dual insurance	404 (32.6)	42 (15.8)		70 (37.6)		48 (20.7)		233 (44.2)		11 (37.9)	
Uninsured	111 (9.0)	36 (13.5)		31 (16.7)		21 (9.1)		19 (3.6)		4 (13.8)	
Prefer not to answer	68 (5.5)	37 (13.9)		5 (2.7)		0		21 (4.0)		5 (17.2)	
Internet access^b^											
Internet access at home	881 (71.0)	141 (53.0)		115 (61.8)		176 (75.9)		430 (81.6)		19 (65.5)	
Desktop	855 (69.0)	132 (49.6)		115 (61.8)		172 (74.1)		418 (79.3)		18 (62.1)	
Mobile device	677 (54.6)	95 (35.7)		77 (41.4)		103 (44.4)		390 (74.0)		12 (41.4)	
Tablet	425 (34.3)	38 (14.3)		21 (11.3)		75 (32.33)		286 (54.3)		5 (17.2)	
Years since diagnosis, median (IQR), y	0 (0-3)	0 (0-2)		0 (0-3)		0 (0-3)		0 (0-3)		0 (0-0)	
Anatomic site of cancer diagnosis											
Breast	208 (16.8)	43 (16.2)		18 (9.7)		22 (9.5)		119 (22.6)		6 (20.7)	
Gastrointestinal	310 (25.0)	59 (22.2)		63 (33.8)		57 (24.6)		121 (23.0)		10 (34.5)	
Genitourinary	100 (8.1)	33 (12.4)		5 (2.7)		16 (6.9)		42 (8.0)		4 (13.8)	
Gynecologic oncology	30 (2.4)	10 (3.8)		0		5 (2.2)		14 (2.7)		1 (3.5)	
Lung	420 (33.9)	99 (37.1)		74 (39.8)		107 (46.1)		132 (25.0)		8 (27.5)	
Malignant hematologic	80 (6.5)	14 (5.3)		8 (4.3)		14 (6.0)		44 (8.3)		0	
Other (soft tissue, brain, head and neck, thyroid, unknown primary, melanoma)	92 (7.3)	8 (3.0)		18 (9.7)		11 (4.7)		55 (10.4)		0	
Cancer stage at diagnosis											
0 (In situ)	28 (2.3)	4 (1.5)		0		1 (0.4)		22 (4.2)		1 (3.5)	
1	102 (8.2)	13 (4.9)		10 (5.4)		18 (7.8)		60 (11.4)		1 (3.5)	
2	106 (8.5)	7 (2.6)		6 (3.2)		17 (7.3)		73 (13.9)		3 (10.3)	
3	120 (9.7)	18 (6.8)		12 (6.5)		18 (7.8)		66 (12.5)		6 (20.7)	
4	198 (16.0)	20 (7.5)		10 (5.4)		29 (12.5)		129 (24.5)		10 (34.5)	
Do not know cancer stage	686 (55.3)	204 (76.7)		148 (79.6)		149 (64.2)		177 (33.6)		8 (27.6)	
Cancer-directed treatment^c^										
Receiving active cancer-directed treatment	852 (68.7)	186 (69.9)		125 (67.2)		174 (75.0)		347 (65.8)		20 (69.0)	
Chemotherapy or immunotherapy	756 (61.0)	167 (62.8)		99 (53.2)		153 (65.9)		320 (60.7)		17 (58.6)	
Receiving chemotherapy or immunotherapy treatment every 2-3 wk	704 (93.1)	158 (94.6)		92 (92.9)		146 (95.4)		293 (91.6)		15 (88.2)	
Receiving chemotherapy or immunotherapy treatment every ≥4 wk	52 (6.9)	9 (5.4)		7 (7.1)		7 7 (4.6)		27 (8.4)		2 (11.8)	
Receiving oral chemotherapy, oral hormonal therapy, or endocrine therapy	90 (7.3)	17 (6.4)		2 (1.1)		7 (3.0)		62 (11.8)		2 (6.9)	
Radiotherapy	284 (22.9)	62 (23.3)		61 (32.8)		61 (26.3)		90 (17.1)		10 (34.5)	
Daily radiotherapy	284 (100)	62 (100)		61 (100)		61 (100)		90 (100)		10 (100)	
Surgery	40 (3.2)	5 (1.9)		13 (7.0)		9 (3.9)		12 (2.3)		1 (3.1)	
Participating in clinical trial	106 (8.5)	10 (3.8)		6 (3.2)		14 (6.0)		74 (14.0)		2 (6.9)	

^a^ Defined race and ethnicity self-reported as American Indian or Alaska Native, Native Hawaiian, or multiple races.

^b^ Multiselect question; the number of responses for this variable exceeds the number of participants and response percentages exceed 100%.

^c^ Number of responses and percentages are based on the number of participants who reported receiving that specific active cancer-directed treatment.

Most respondents were married (672 [54.2%]) and living with 1 or more household members (1146 [92.4%]). A higher proportion of Black respondents (230 of 266 [86.5%]), Asian respondents (166 of 186 [89.2%]), and Latinx respondents (188 of 232 [81.0%]) lived with a household member 18 years of age or younger; a higher proportion of Latinx respondents (175 of 232 [75.4%]) lived with a household member 65 years of age or older. Most respondents (1016 [81.9%]) reported annual household incomes of less than $100 000 US dollars, which was more common among Black respondents (252 of 266 [94.7%]). Most respondents had a bachelor’s degree or other professional degree (629 [50.7%]), which was more common among Asian respondents (126 of 186 [67.7%]).

Many respondents reported full-time employment (244 [19.7%]) or part-time employment (56 [4.5%]); a higher proportion of Black respondents were unemployed (106 of 266 [39.8%]). Most respondents had insurance (1110 [89.5%]). A higher proportion of Latinx respondents had private insurance or dual insurance coverage (176 of 232 [75.9%]). Most respondents had home internet access (894 [72.1%]), which was most common among White respondents (434 of 527 [82.4%]).

### Clinical Characteristics

The median time to cancer diagnosis was 0 years (IQR, 0-3 years). Lung cancer was the most common diagnosis (420 [33.9%]) (eTable 1 in the Supplement). Most respondents (686 [55.3%]) did not know their cancer stage at diagnosis, 852 (68.7%) reported receiving cancer-directed therapy, 756 (61.0%) reported receiving chemotherapy or immunotherapy, 284 (22.9%) reported receiving radiotherapy, and 64 (5.2%) were scheduled for surgery. Of the 756 respondents receiving chemotherapy or immunotherapy, 696 (92.1%) reported undergoing treatment every 2 to 3 weeks, and of the 284 respondents receiving radiotherapy, 284 (100.0%) reported undergoing treatment daily. A higher proportion of White respondents were enrolled in a clinical trial (104 of 527 [19.7%]).

### Modifications in Care Due to the COVID-19 Pandemic

A higher proportion of Black respondents (201 of 266 [75.6%]), Latinx respondents (186 of 232 [80.2%]), and respondents of other race or ethnicity (22 of 29 [75.9%]) experienced modifications in care, including delayed clinic visits, laboratory tests, and imaging and a change in location of care compared with White participants (301 of 527 [57.1%]) (Table 2). A higher proportion of Black respondents (197 of 201 [98.0%]) than White respondents (253 of 301 [84.1%]) who experienced modifications in care reported that the modifications were requested by the clinic or physician.

**Table 2.** Modifications in Care Due to the COVID-19 Pandemic by Race and Ethnicity in a National Survey Study of 1240 Adults With Cancer in the US From September 1, 2020, to January 12, 2021

Variable	Participants, No./total No. (%)					
	Total (N = 1240)	African American or Black (n = 266)	Asian (n=186)	Hispanic or Latinx (n = 232)	White (n = 527)	Other (n = 29)^a^
Modification in care, No. (%)						
None	434 (35.0)	65 (24.4)	90 (48.4)	46 (19.8)	226 (42.9)	7 (24.1)
≥1	806 (65.0)	201 (75.6)	96 (51.6)	186 (80.2)	301 (57.1)	22 (75.9)
Type of modification in care^b^						
Delay or cancellation of follow-up clinic visit	615/806 (76.3)	193/201 (96.0)	87/96 (90.6)	117/186 (62.9)	196/301 (65.1)	22/22 (100.0)
Delay or cancellation of laboratory visit	554/806 (68.7)	192/201 (95.5)	87/96 (90.6)	105/186 (56.4)	148/301 (49.2)	22/22 (100.0)
Delay or cancellation of imaging	495/806 (61.4)	120/201 (59.7)	75/96 (78.1)	106/186 (57.0)	178/301 (59.1)	16/22 (80.7)
Change in treatment schedule	70/806 (8.7)	28/201 (13.9)	10/96 (10.4)	14/186 (7.5)	16/301 (5.3)	2/22 (9.1)
New medication	46/806 (5.7)	12/201 (6.0)	6/96 (6.3)	9/186 (4.8)	18/301 (6.0)	1/22 (4.5)
Location of care change	44/806 (5.5)	16/201 (8.0)	3/96 (3.1)	11/186 (5.9)	9/301 (3.0)	5/22 (22.7)
Reason for modification in care						
Requested by clinic or physician	737/806 (91.4)	197/201 (98.0)	93/96 (96.9)	177/186 (95.2)	253/301 (84.1)	17/22 (77.3)
Do not know	18/806 (2.2)	1/201 (0.5)	0	6/186 (3.2)	8/301 (2.7)	3/22 (13.6)
Self-requested	51/806 (6.3)	3/201 (1.5)	3/96 (3.1)	3/186 (1.6)	40/301 (13.3)	2/22 (9.1)

^a^ Defined race and ethnicity self-reported as American Indian or Alaska Native, Native Hawaiian, or multiple races.

^b^ Multiselect question; response percentages for each option (ie, specific type of modification) when summed will exceed 100%.

**Figure 1.**
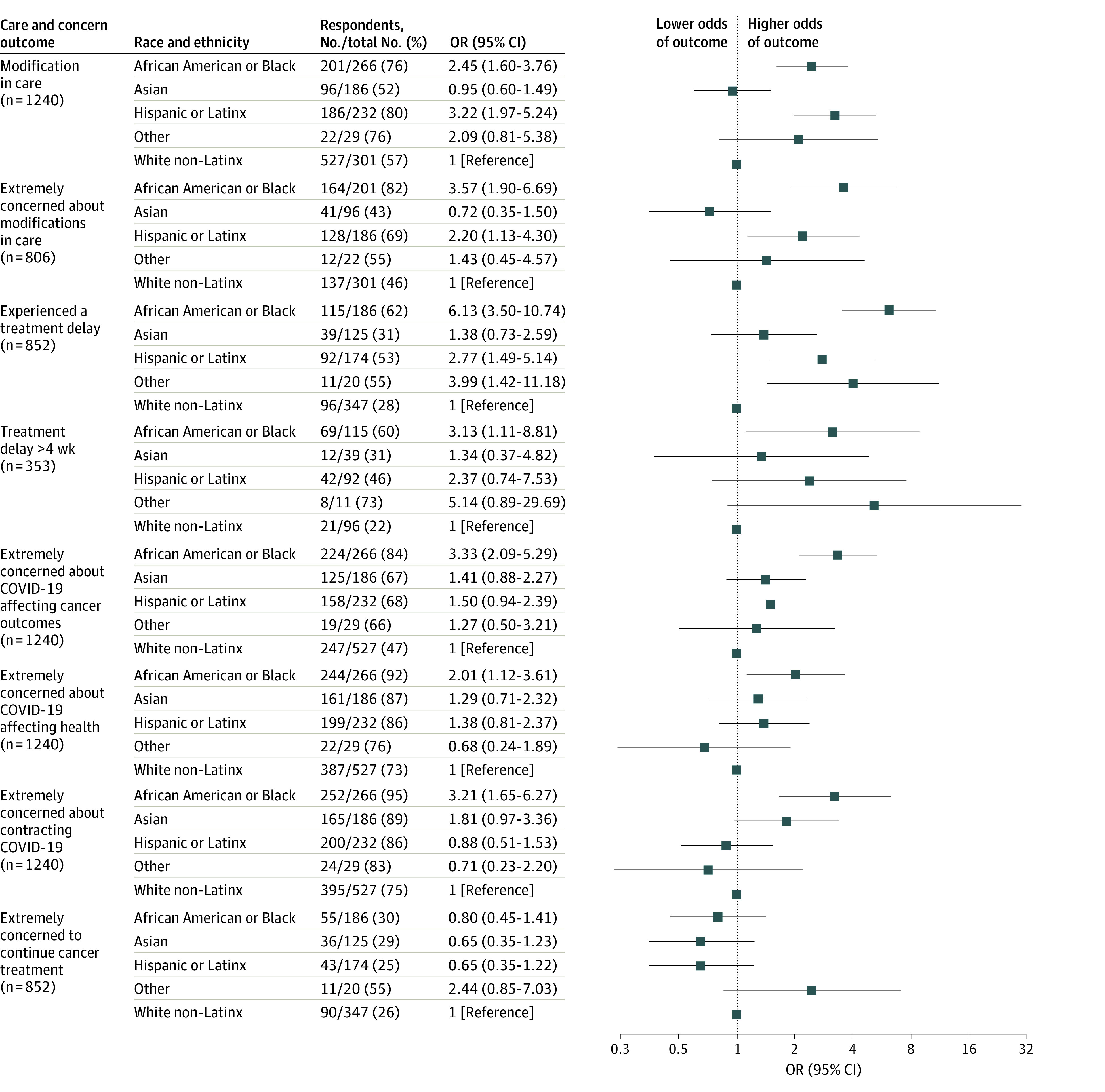
Modifications in Cancer Care, Treatment Delays, and Concerns Regarding Health by Race and Ethnicity The reference group for all comparisons is White non-Latinx respondents. Models were adjusted for a priori selected sociodemographic (gender identity, age, educational level, income, insurance status, and place of residence) and clinical variables (cancer diagnosis and stage). The other race category comprises American Indian or Alaska Native, Native Hawaiian, and respondents of multiple races. OR indicates odds ratio.

Compared with White respondents, Black respondents (OR, 2.45 [95% CI, 1.60-3.76]) and Latinx respondents (OR, 3.22 [95% CI, 1.97-5.24]) had greater odds of experiencing modifications in care and greater odds of reporting extreme concerns that the modifications would worsen their cancer outcomes (Black respondents: OR, 3.57 [95% CI, 1.90-6.69]; Latinx respondents: OR, 2.20 [95% CI 1.13-4.30]) (Figure 1).

Among those receiving cancer-directed treatment, Black respondents (OR, 6.13 [95% CI, 3.50-10.74]), Latinx respondents (OR, 2.77 [95% CI, 1.49-5.14]), and respondents of other race or ethnicity (OR, 3.99 [95% CI, 1.42-11.18]) had greater odds of experiencing involuntary treatment delays (Figure 1). Compared with White respondents, Black respondents had greater odds of experiencing treatment delays that exceeded 4 weeks (OR, 3.13 [95% CI, 1.11-8.81]) or experiencing indefinite treatment cancellation (OR, 2.15 [95% CI, 1.52-2.78]).

### Concerns Regarding Health Outcomes

Compared with White respondents, Black respondents had greater odds of extreme concerns that the COVID-19 pandemic would affect cancer outcomes (OR, 3.33 [95% CI, 2.09-5.29]) and other health outcomes (OR, 2.01 [95% CI, 1.12-3.61]) as well as greater odds of extreme concerns about contracting SARS-CoV-2 (OR, 3.21 [95% CI, 1.65-6.27]) (Figure 1). There were no differences by race and ethnicity in the odds of concerns for continuing cancer treatment.

### Experiences and Concerns Regarding Adverse Social and Economic Effects

**Figure 2.**
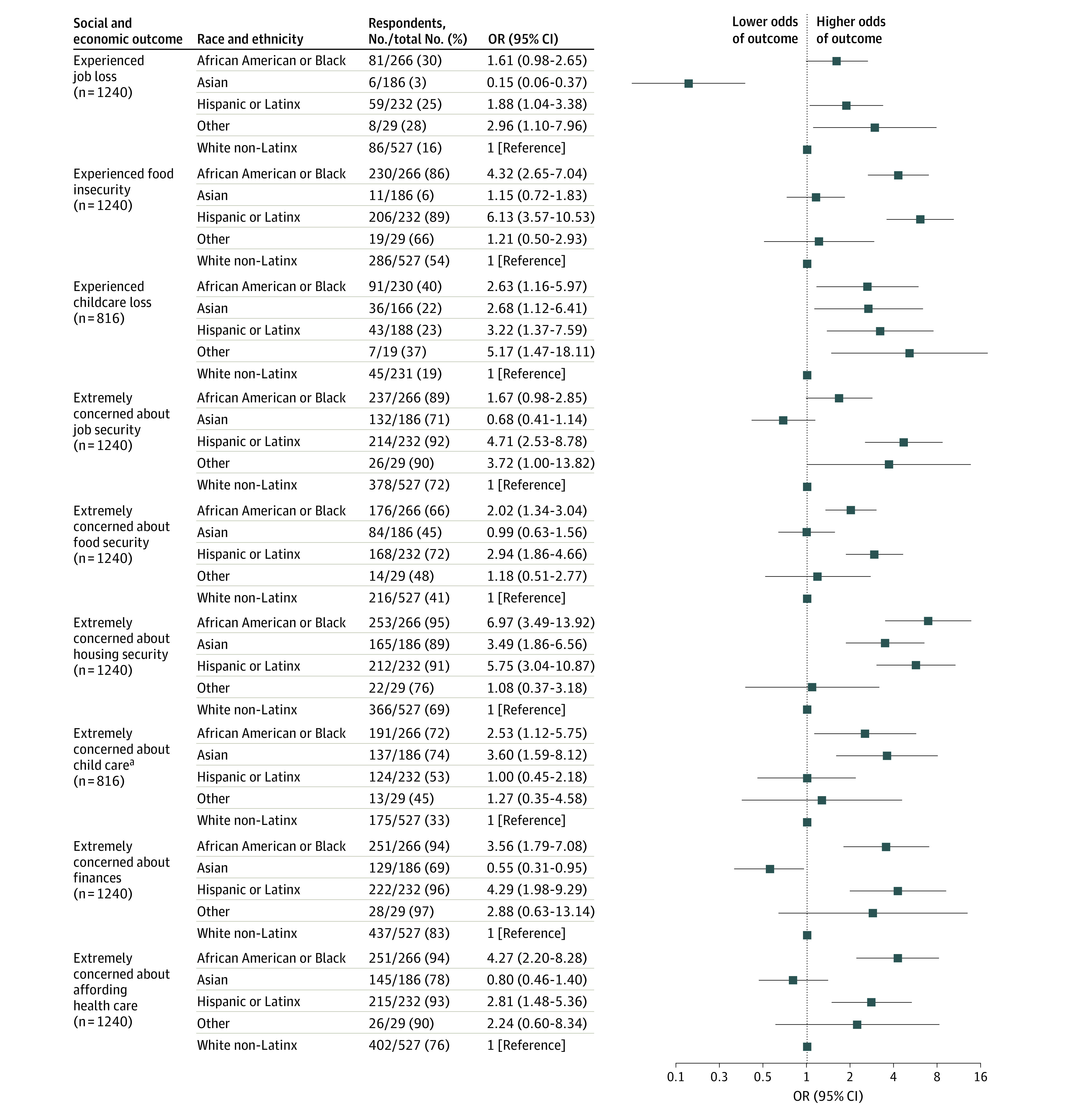
Experiences and Concerns Regarding Adverse Social and Economic Effects by Race and Ethnicity The reference group for all comparisons is White non-Latinx respondents. Models were adjusted for a priori selected sociodemographic (gender identity, age, educational level, income, insurance status, and place of residence) and clinical variables (cancer diagnosis and stage). The other race category comprises American Indian or Alaska Native, Native Hawaiian, and respondents of multiple races. OR indicates odds ratio. ^a^In the model assessing concerns about child care, 1 iteration of the multiple imputation for unknown cancer stage generated a structural positivity violation in which there were no cases among stage 0. This imputation was excluded, and the results comprise 99 iterations.

Compared with White respondents, Latinx respondents (OR, 1.88 [95% CI, 1.04-3.38) and respondents of other race or ethnicity (OR, 2.96 [95% CI, 1.10-7.96]) had greater odds of losing their job (Figure 2). Black respondents (OR, 4.32 [95% CI, 2.65-7.04]) and Latinx respondents (OR, 6.13 [95% CI, 3.57-10.53]) had greater odds than White respondents of food insecurity. Among respondents with children, all non-White groups had greater odds than White respondents of childcare loss (Black respondents: OR, 2.63 [95% CI, 1.16-5.97]; Asian respondents: OR, 2.68 [95% CI, 1.12-6.41]; Latinx respondents: OR, 3.22 [95% CI, 1.37-7.59]; and respondents of other race or ethnicity: OR, 5.17 [95% CI, 1.47-18.11]).

### Extreme Concerns Regarding Adverse Social and Economic Effects

Latinx respondents had greater odds than White respondents of extreme concerns regarding job security (OR, 4.71 [95% CI, 2.53-8.78]) (Figure 2). Black respondents (OR, 2.02 [95% CI, 1.34-3.04]) and Latinx respondents (OR, 2.94 [95% CI, 1.86-4.66]) had greater odds than White respondents of extreme concerns regarding food security. Black respondents (OR, 6.97 [95% CI, 3.49-13.92]), Asian respondents (OR, 3.49 [95% CI, 1.86-6.56]), and Latinx respondents (OR, 5.75 [95% CI, 3.04-10.87]) had greater odds than White respondents of extreme concerns regarding housing security. Among those with children, Black respondents (OR, 2.53 [95% CI, 1.12-5.75]) and Asian respondents (OR, 3.60 [95% CI, 1.59-8.12]) had greater odds than White respondents of extreme concerns regarding child care. Black respondents (OR, 3.56 [95% CI, 1.79-7.08]) and Latinx respondents (OR, 4.29 [95% CI, 1.98-9.29]) had greater odds than White respondents of extreme concerns regarding overall finances, as well as affordability of health care (Black respondents: OR, 4.27 [95% CI, 2.20-8.28]; Latinx respondents OR, 2.81 [95% CI, 1.48-5.36]).

### Trust in Sources of COVID-19 Information

**Figure 3.**
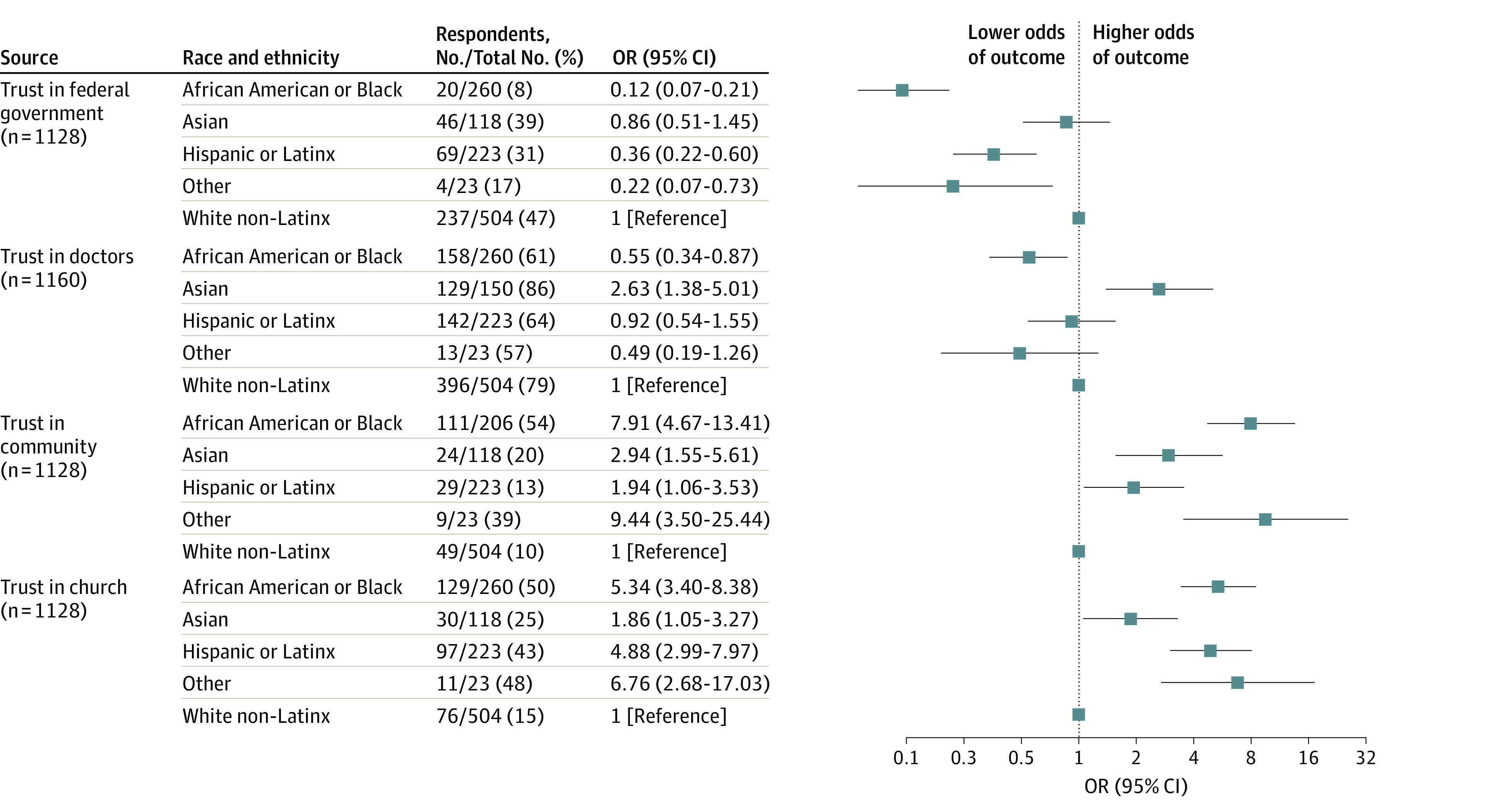
Trust in Sources for COVID-19 Information by Race and Ethnicity The reference group for all comparisons is White non-Latinx respondents. Models were adjusted for a priori selected sociodemographic (gender identity, age, educational level, income, insurance status, and place of residence) and clinical variables (cancer diagnosis and stage). The other race category comprises American Indian or Alaska Native, Native Hawaiian, and respondents of multiple races. OR indicates odds ratio.

Black respondents (OR, 0.12 [95% CI, 0.07-0.21]), Latinx respondents (OR, 0.36 [95% CI, 0.22-0.60]), and respondents of other race or ethnicity (OR, 0.22 [95% CI, 0.07-0.73]) had lower odds of trust than White respondents in the sources of COVID-19 information from the federal government (Figure 3). Compared with White respondents, Black respondents had lower odds of trust in physicians for COVID-19 information (OR, 0.55 [95% CI, 0.34-0.87]), and Asian respondents had higher odds of trust in physicians for COVID-19 information (OR, 2.63 [95% CI, 1.38-5.01]). Compared with White respondents, all non-White respondents had greater odds of trust in their community for COVID-19 information (Black respondents: OR, 7.91 [95% CI, 4.67-13.41]; Asian respondents: OR, 2.94 [95% CI, 1.55-5.61]; Latinx respondents: OR, 1.94 [95% CI, 1.06-3.53]; and respondents of other race or ethnicity: OR, 9.44 [95% CI, 3.50-25.44]) and greater odds of trust in church for COVID-19 information (Black respondents: OR, 5.34 [95% CI, 3.40-8.38]; Asian respondents: OR, 1.86 [95% CI,1.05-3.27]; Latinx respondents: OR, 4.88 [95% CI, 2.99-7.97]; and respondents of other race or ethnicity: OR, 6.76 [95% CI, 2.68-17.03]).

### Sensitivity Analyses

Black respondents had 68% lower odds of knowing their cancer stage than White respondents (OR, 0.32 [95% CI, 0.21-0.49]), and Latinx respondents had 47% lower odds of knowing their cancer stage than White respondents (OR 0.53 [95% CI, 0.36-0.76]). In a sensitivity analysis in which unknown cancer stage was coded as a separate category within the cancer stage, ORs were similar in direction but varied in magnitude compared with those of the primary analysis (eTables 2-6 in the Supplement). Odds ratios for the complete-case analyses were consistent with the primary analysis; however, the large 95% CIs reflect the limited power.

## Discussion

In this survey study of US adults with cancer, compared with White respondents, Black and Latinx respondents had 3 times the odds of experiencing modifications in their cancer care and extensive cancer treatment delays and greater odds of adverse social and economic effects and concerns regarding future adverse social and economic effects of the COVID-19 pandemic.

Owing to a combination of structural, economic, and socioenvironmental factors associated with systemic racism, prepandemic disparities persist in access to and timely receipt of cancer care among Black and Latinx adults. The COVID-19 pandemic heightened national awareness of structural barriers, including inadequacies in the health care system, that are associated with disparities in cancer care delivery. In this study, similar to 2 prior single-institution studies among patients with breast and prostate cancer,^16,17^ Black and Latinx adults experienced not only more delays but 6 times and nearly 3 times the odds, respectively, of cancer treatment delays that exceeded 4 weeks compared with White adults. The findings in this study, comprising mostly participants receiving treatment every 2 to 3 weeks and/or daily radiotherapy, suggest that 1 or more courses (or cycles) of treatment were delayed or canceled indefinitely. The long-term effects of 4 weeks’ or more delay on cancer outcomes remains unknown, yet in 1 meta-analysis, each month that cancer treatment was delayed was associated with a 10% increase in the risk of death.^25^ Although it is possible that delays in care may have been associated with fear of seeking care during the pandemic, there were no differences in concerns regarding continuation of cancer treatment by race and ethnicity.

Implicit bias and systemic racism have impaired educational, employment, and housing opportunities for Black adults for decades. These inequitable impairments are associated with prepandemic disparities, in which Black adults had half the access to health care, more food insecurity, and more poverty than White adults.^10,26,27,28^ The COVID-19 pandemic exacerbated these preexisting disparities. Between March and April 2020, for example, unemployment rates tripled and were disproportionately higher among Black and Latinx adults compared with adults of other races and ethnicities.^15,29,30^ In this study, conducted more than 4 months later, Black and Latinx adults experienced disparate adverse social and economic effects, such as unemployment among Latinx adults and food insecurity among both Black and Latinx adults.^31,32,33,34,35^

Although worsening mental and emotional health among patients with cancer has been described,^36,37^ in this study, Black and Latinx adults had greater odds than White non-Latinx adults of reporting extreme concerns that the COVID-19 pandemic and its associated delays in care would worsen their cancer outcomes. In contrast to results previously reported by survivors of breast cancer, which show no disparities in concerns regarding health outcomes by race and ethnicity,^38^ this study included participants with all cancer diagnoses, most of whom were undergoing active cancer treatment at the time of the survey. Black and Latinx adults also had greater odds of extreme concerns regarding future adverse social and economic effects, including food insecurity, housing insecurity, overall financial instability, and unaffordability of cancer care. These findings further support the worsening health and well-being of Black and Latinx adults during the ongoing pandemic.^39^

Trust is an essential component associated with an individual’s understanding of information and willingness to act,^40,41^ and it varies by race and ethnicity.^42,43^ In this study, consistent with others,^42^ Black and Latinx adults had less trust in the federal government, and Black adults had less trust in physicians for COVID-19 information. Adults from non-White groups represented in this study had greater odds of trust than White adults in community and church or faith-based organizations for COVID-19 information. Increased vaccination rates among Black adults through efforts led predominantly by community and church or faith-based organizations^44^ support the importance of partnering with sources of trust to improve health outcomes for individual groups. These findings should be explored as opportunities to overcome structural factors associated with longstanding cancer disparities.

### Limitations

Our study had some limitations. The response rate could be an overestimation because it was calculated among those who directly received the survey link and not among those who may have seen the survey on social media. Individuals may have responded if they had a negative experience exacerbated by the pandemic, resulting in recall bias that may affect our results. Selection bias due to online survey access may have restricted participation to adults with internet access. Respondents had a high educational level, possibly underestimating results among populations with a lower educational level. The low number of self-identified Native Hawaiian, Alaska Native, and American Indian participants prevented statistical analysis of these groups in distinct categories. Our pooled associations (ie, the other race and ethnicity category) may not be the same for each of these groups.

The date of cancer diagnosis was not an inclusion criterion. Therefore, some respondents may have been outside the time period of treatment and might not have experienced modifications in cancer care. However, the median time of diagnosis was less than 1 year, and most participants reported receiving active cancer treatment at the time of the survey. Because we did not conduct a prepandemic and postpandemic evaluation, our results cannot discern whether the disparities reported were worse than those before the pandemic. Delays in care, such as those reported in this study, were due to a complex interplay between cancer diagnosis, cancer stage, treatment, and local COVID-19 infection rates. It is possible, therefore, that inaccurate self-reported cancer diagnosis and stage may have resulted in misclassifications that may have affected our results. Studies show that Black and Latinx adults have more advanced cancer stages at diagnosis than any other racial and ethnic group.^45,46^ In this study, however, a high proportion of respondents did not know their cancer stage, which was differential by race and ethnicity, with Black and Latinx adults having lower odds of knowing their cancer stage than White non-Latinx adults. In our primary analyses, we imputed unknown cancer stages, given studies that show that cancer stage could be missing at random, conditional on race, gender identity, age, income, insurance, and educational level,^47,48,49^ and we included the other clinical variables in the multiple imputation models.^50,51,52,53,54^ In sensitivity analyses, we analyzed unknown cancer stage as a distinct value, and we also conducted a complete-case analysis among participants who reported a known cancer stage. Although the magnitude of the ORs in these analyses varied, the direction of the association was consistent. However, because it is impossible to ascertain whether data are missing at random, our results, including multiple imputed cancer stage for unknown values, should be interpreted within this limitation.

## Conclusions

In this survey study of US adults with cancer, Black and Latinx adults experienced extensive cancer care disparities, including greater delays in cancer treatment, adverse social and economic effects, and concerns regarding future adverse social and economic effects, compared with White adults during the COVID-19 pandemic. Urgent efforts are needed to address these disparities among Black and Latinx adults with cancer.

## Notes

Supplement. **eFigure.** Flow Chart of Survey Population and Respondents in a National Survey Study of 1240 Adults With Cancer in the United States From September 1, 2020, to January 12, 2021 **eTable 1.** Detailed Anatomic Site of Cancer Diagnoses of the Study Participants by Race and Ethnicity in a National Survey Study of 1240 Adults With Cancer in the United States From September 1, 2020, to January 12, 2021 **eTable 2.** Sensitivity Analysis of Modifications in Cancer Care, Concern, and Treatment Delays by Race and Ethnicity in a National Survey Study of 1240 Adults With Cancer in the United States From September 1, 2020, to January 12, 2021 **eTable 3.** Sensitivity Analysis for Concern Regarding Health Outcomes by Race and Ethnicity in a National Survey Study of 1240 Adults With Cancer in the United States From September 1, 2020, to January 12, 2021 **eTable 4.** Sensitivity Analysis for Social and Economic Impacts by Race and Ethnicity in a National Survey Study of 1240 Adults With Cancer in the United States From September 1, 2020, to January 12, 2021 **eTable 5.** Sensitivity Analysis for Concern Regarding Social and Economic Impacts by Race and Ethnicity in a National Survey Study of 1240 Adults With Cancer in the United States From September 1, 2020, to January 12, 2021 **eTable 6.** Sensitivity Analysis for Sources of Trust by Race and Ethnicity for COVID-19 Information in a National Survey Study of 1240 Adults With Cancer in the United States From September 1, 2020, to January 12, 2021 **eAppendix.** Survey Invitation and Survey
