# Supplementary material for: Racial and Ethnic Disparities in Cancer Care During the COVID-19 Pandemic
Source: JAMA Netw Open. 2022 Jul 14;5(7):e2222009. doi: 10.1001/jamanetworkopen.2022.22009 (PMC9284331; doi:10.1001/jamanetworkopen.2022.22009)
Supplement: Supplement. — eFigure. Flow Chart of Survey Population and Respondents in a National Survey Study of 1240 Adults With Cancer in the United States From September 1, 2020, to January 12, 2021 eTable 1. Detailed Anatomic Site of Cancer Diagnoses of the Study Participants by Race and Ethnicity in a National Survey Study of 1240 Adults With Cancer in the United States From September 1, 2020, to January 12, 2021 eTable 2. Sensitivity Analysis of Modifications in Cancer Care, Concern, and Treatment Delays by Race and Ethnicity in a National Survey Study of 1240 Adults With Cancer in the United States From September 1, 2020, to January 12, 2021 eTable 3. Sensitivity Analysis for Concern Regarding Health Outcomes by Race and Ethnicity in a National Survey Study of 1240 Adults With Cancer in the United States From September 1, 2020, to January 12, 2021 eTable 4. Sensitivity Analysis for Social and Economic Impacts by Race and Ethnicity in a National Survey Study of 1240 Adults With Cancer in the United States From September 1, 2020, to January 12, 2021 eTable 5. Sensitivity Analysis for Concern Regarding Social and Economic Impacts by Race and Ethnicity in a National Survey Study of 1240 Adults With Cancer in the United States From September 1, 2020, to January 12, 2021 eTable 6. Sensitivity Analysis for Sources of Trust by Race and Ethnicity for COVID-19 Information in a National Survey Study of 1240 Adults With Cancer in the United States From September 1, 2020, to January 12, 2021 eAppendix. Survey Invitation and Survey [file jamanetwopen-e2222009-s001.pdf]

Supplementary Online Content

Patel MI, Ferguson JM, Castro E, et al. Racial and ethnic disparities in cancer care during the COVID-19 pandemic. *JAMA Netw Open.* 2022;5(7):e2222009. doi:10.1001/jamanetworkopen.2022.22009

eFigure. Flow Chart of Survey Population and Respondents in a National Survey Study of 1240 Adults With Cancer in the United States From September 1, 2020, to January 12, 2021
eTable 1. Detailed Anatomic Site of Cancer Diagnoses of the Study Participants by Race and Ethnicity in a National Survey Study of 1240 Adults With Cancer in the United States From September 1, 2020, to January 12, 2021
eTable 2. Sensitivity Analysis of Modifications in Cancer Care, Concern, and Treatment Delays by Race and Ethnicity in a National Survey Study of 1240 Adults With Cancer in the United States From September 1, 2020, to January 12, 2021
eTable 3. Sensitivity Analysis for Concern Regarding Health Outcomes by Race and Ethnicity in a National Survey Study of 1240 Adults With Cancer in the United States From September 1, 2020, to January 12, 2021
eTable 4. Sensitivity Analysis for Social and Economic Impacts by Race and Ethnicity in a National Survey Study of 1240 Adults With Cancer in the United States From September 1, 2020, to January 12, 2021
eTable 5. Sensitivity Analysis for Concern Regarding Social and Economic Impacts by Race and Ethnicity in a National Survey Study of 1240 Adults With Cancer in the United States From September 1, 2020, to January 12, 2021
eTable 6. Sensitivity Analysis for Sources of Trust by Race and Ethnicity for COVID-19 Information in a National Survey Study of 1240 Adults With Cancer in the United States From September 1, 2020, to January 12, 2021
eAppendix. Survey Invitation and Survey

This supplementary material has been provided by the authors to give readers additional information about their work.

eFigure. Flow Chart of Survey Population and Respondents in a National Survey Study of 1240 Adults With Cancer in the United States From September 1, 2020, to January 12, 2021

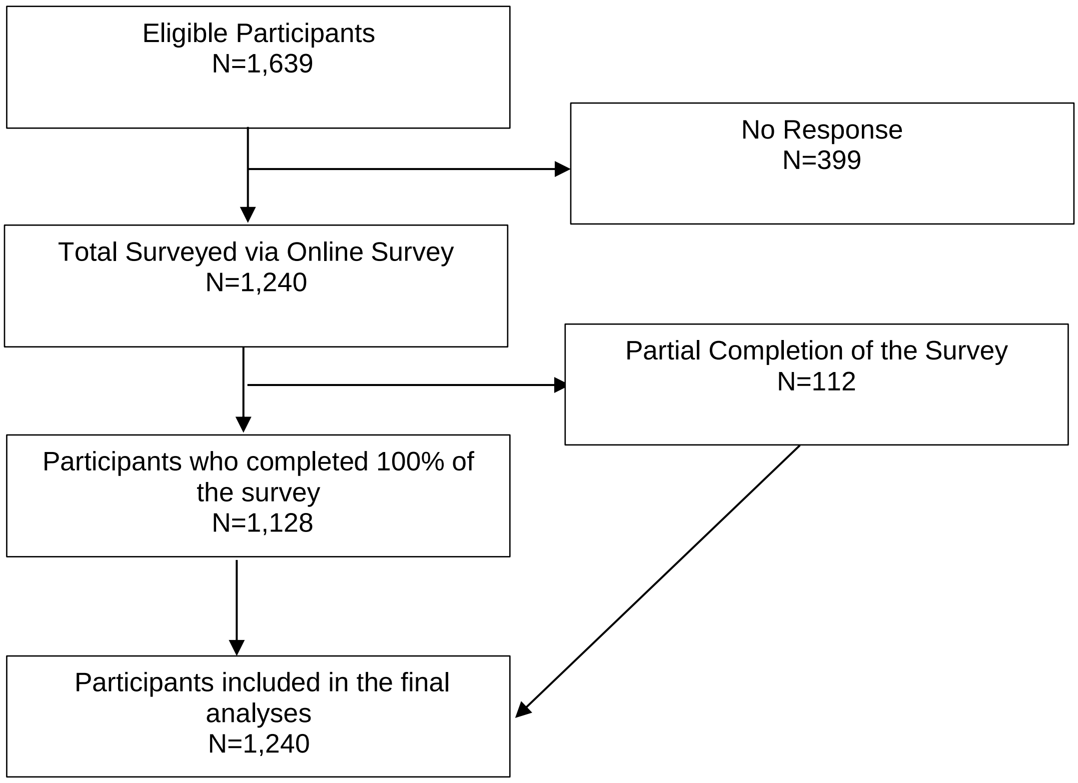

© 2022 Patel MI et al. *JAMA Network Open.*

eTable 1. Detailed Anatomic Site of Cancer Diagnoses of the Study Participants by Race and Ethnicity in a National Survey Study of 1240 Adults With Cancer in the United States From September 1, 2020, to January 12, 2021

Cancer Diagnoses	Participants (1240)	African American or Black (266 [21.5%])	Asian (186 [15%])	Hispanic or Latinx (232 [18.7%])	Other[a] (29 [2.3%])	White (527 [42.5%])
Bladder Cancer	4 (0.3)	3 (1.1)	0 (0.0)	0 (0.0)	0 (0.0)	1 (0.2)
Brain	10 (0.8)	0 (0.0)	4 (2.2)	2 (0.9)	0 (0.0)	4 (0.8)
Breast	208 (16.8)	43 (16.2)	18 (9.7)	22 (9.5)	6 (20.7)	119 (22.6)
Cervical	2 (0.2)	2 (0.8)	0 (0.0)	0 (0.0)	0 (0.0)	0 (0.0)
Colon	184 (14.8)	34 (12.8)	27 (14.5)	39 (16.8)	7 (24.1)	78 (14.9)
Esophageal	28 (2.3)	9 (3.4)	10 (5.4)	4 (1.7)	0 (0.0)	5 (0.9)
Gallbladder	2 (0.2)	0 (0.0)	0 (0.0)	1 (0.4)	0 (0.0)	5 (0.9)
Head and Neck	6 (0.5)	4 (1.5)	0 (0.0)	1 (0.4)	0 (0.0)	1 (0.2)
Kidney	12 (0.9)	3 (1.1)	0 (0.0)	4 (1.7)	3 (10.3)	2 (0.4)
Leukemia	30 (2.4)	4 (1.5)	4 (2.2)	7 (3.0)	0 (0.0)	15 (2.9)
Liver	6 (0.5)	4 (1.5)	0 (0.0)	0 (0.0)	0 (0.0)	2 (0.4)
Lung	420 (33.9)	99 (37.2)	74 (39.8)	107 (46.1)	8 (27.6)	132 (25.1)
Lymphoma	38 (3.1)	8 (3.0)	4 (2.2)	5 (2.2)	0 (0.0)	21 (3.9)
Melanoma	20 (1.6)	0 (0.0)	2 (1.1)	2 (0.9)	0 (0.0)	16 (3.0)
Multiple Myeloma	10 (0.8)	2 (0.8)	0 (0.0)	2 (0.9)	0 (0.0)	6 (1.1)
Myelodysplastic Syndrome	2 (0.2)	0 (0.0)	0 (0.0)	0 (0.0)	0 (0.0)	2 (0.4)
Unknown primary	38 (3.1)	0 (0.0)	4 (2.2)	6 (2.6)	0 (0.0)	28 (5.3)
Ovarian	18 (1.5)	4 (1.5)	0 (0.0)	3 (1.3)	1 (3.5)	10 (1.9)
Pancreas	18 (1.5)	2 (0.8)	2 (1.1)	4 (1.7)	0 (0.0)	10 (1.9)
Prostate	82 (6.6)	27 (10.2)	5 (2.7)	12 (5.2)	1 (3.5)	37 (7.0)
Rectal	54 (4.4)	7 (2.6)	12 (6.5)	9 (3.9)	2 (6.9)	24 (4.6)
Soft Tissue	12 (0.9)	2 (0.8)	6 (3.2)	0 (0.0)	0 (0.0)	4 (0.8)
Stomach	16 (1.3)	3 (1.1)	12 (6.5)	0 (0.0)	1 (3.5)	0 (0.0)
Testicular	2 (0.2)	0 (0.0)	0 (0.0)	0 (0.0)	0 (0.0)	2 (0.4)
Thyroid	6 (0.5)	2 (0.8)	2 (1.1)	0 (0.0)	0 (0.0)	2 (0.4)
Uterine	10 (0.8)	4 (1.5)	0 (0.0)	2 (0.9)	2 (0.9)	4 (0.8)

[a]Other is defined as race and ethnicity which is self-reported as Native Hawaiian/American Indian/Alaska Native/Multiple Race.

© 2022 Patel MI et al. *JAMA Network Open.*

eTable 2. Sensitivity Analysis of Modifications in Cancer Care, Concern, and Treatment Delays by Race and Ethnicity in a National Survey Study of 1240 Adults With Cancer in the United States From September 1, 2020, to January 12, 2021[a]

	African American or Black				Asian				Hispanic or Latinx				Other[b]				White			
	Total Response No.	No. (%)	OR	95% CI	Total Response No.	No. (%)	OR	95% CI	Total Response No.	No. (%)	OR	95% CI	Total Response No.	No. (%)	OR	95% CI	Total Response No.	No. (%)	OR	95% CI
**Experienced a modification in care**																				
Model A N=1240	266	201 (75.6)	2.45	1.60-3.76	186	96 (51.6)	0.95	0.60-1.49	232	186 (80.2)	3.22	1.97-5.24	29	22 (75.9)	2.09	0.81-5.38	527	301 (57.1)	1.00	--
Model B N=1240	266	201 (75.6)	2.40	1.57-3.69	186	96 (51.6)	0.94	0.60-1.48	232	186 (80.2)	3.18	1.95-5.17	29	22 (75.9)	2.04	0.80-5.23	527	301 (57.1)	1.00	--
Model C N=554	62	42 (67.7)	4.44	2.21-8.94	38	19 (50.0)	1.69	0.73-3.90	83	55 (66.3)	2.49	1.35-4.61	21	17 (81.0)	5.37	1.53-18.86	350	168 (48.0)	1.00	--
**Extremely concerned that modification in care will worsen cancer outcomes**																				
Model A N=806	201	164 (81.6)	3.57	1.90-6.69	96	41 (42.7)	0.72	0.35-1.50	186	128 (68.8)	2.20	1.13-4.30	22	12 (54.5)	1.43	0.45-4.57	301	137(45.5)	1.00	--
Model B N=806	201	164 (81.6)	3.04	1.61-5.75	96	41 (42.7)	0.63	0.30-1.31	186	128 (68.8)	2.10	1.06-4.14	22	12 (54.5)	1.61	0.50-5.15	301	137(45.5)	1.00	--
Model C N=301	42	18 (42.9)	4.21	1.41-12.51	19	3 (15.8)	2.34	0.42-13.00	55	18 (32.7)	2.57	0.97-6.79	17	7 (41.2)	6.72	1.52-29.65	168	32 (19.0)	1.00	--
**Experienced a treatment delay (N=852)**																				
Model A N=852	186	115 (61.8)	6.13	3.50-10.74	125	39 (31.2)	1.38	0.73-2.59	174	92 (52.9)	2.77	1.49-5.14	20	11(55.0)	3.99	1.42-11.18	347	96 (27.7)	1.00	--
Model B N=852	186	115 (61.8)	5.82	3.33-10.19	125	39 (31.2)	1.28	0.68-2.42	174	92 (52.9)	2.73	1.47-5.08	20	11(55.0)	3.98	1.42-11.13	347	96 (27.7)	1.00	--
Model C N=332	40	24 (60.0)	9.90	3.92-24.99	22	6 (27.3)	3.95	1.15-13.56	50	24 (48.0)	7.32	2.97-18.01	14	10(71.4)	14.02	3.28-59.95	206	31 (15.0)	1.00	--
**Experienced more than 4 weeks treatment delay or indefinite cancellation (N=353)**																				
Model A N=353	115	69 (60.0)	3.13	1.11-8.81	39	12 (30.8)	1.34	0.37-4.82	92	42 (45.7)	2.37	0.74-7.53	11	8 (72.7)	5.14	0.89-29.69	96	21 (21.9)	1.00	--
Model B N=353	115	69 (60.0)	3.15	1.13-8.79	39	12 (30.8)	1.34	0.38-4.72	92	42 (45.7)	2.31	0.73-7.25	11	8 (72.7)	5.73	0.99-32.97	96	21 (21.9)	1.00	--
Model C N=95	24	13 (54.2)	3.33	0.55-20.12	6	3 (50.0)	4.34	0.25-76.04	24	11 (45.8)	7.12	1.09-46.40	10	8 (80.0)	40.33	1.64-994.30	31	13 (41.9)	1.00	--

[a]Model A is regression analysis output with unknown cancer stage values imputed using Multiple Imputation. Model B is sensitivity analysis output with unknown stage values accounted for as a separate category within the cancer stage variable. Model C is complete case analysis output for the 554 participants who reported a known cancer stage. Referent group for all comparisons is White. Models were adjusted for apriori selected sociodemographic (gender, age, education, income, insurance status, place of residence) and clinical variables (cancer diagnosis, stage). Abbreviations: OR, odds ratio; CI, confidence interval.

[b]Other race category comprises American Indian/Alaska Native, Native Hawaiian, and Multiple Race.

© 2022 Patel MI et al. *JAMA Network Open.*

eTable 3. Sensitivity Analysis for Concern Regarding Health Outcomes by Race and Ethnicity in a National Survey Study of 1240 Adults With Cancer in the United States From September 1, 2020, to January 12, 2021[a]

	African American or Black				Asian				Hispanic or Latinx				Other[b]				White			
	Total Response No.	No. (%)	OR	95% CI	Total Response No.	No. (%)	OR	95% CI	Total Response No.	No. (%)	OR	95% CI	Total Response No.	No. (%)	OR	95% CI	Total Response No.	No. (%)	OR	95% CI
**Extremely concerned about the COVID-19 pandemic affecting cancer outcomes**																				
Model A N=1240	266	224 (84.2)	3.33	2.09-5.29	186	125 (67.2)	1.41	0.88-2.27	232	158 (68.1)	1.50	0.94-2.39	29	19 (65.5)	1.27	0.50-3.21	527	247 (46.9)	1.00	--
Model B N=1240	266	224 (84.2)	2.98	1.87-4.73	186	125 (67.2)	1.25	0.78-2.00	232	158 (68.1)	1.42	0.89-2.27	29	19 (65.5)	1.32	0.52-3.36	527	247 (46.9)	1.00	--
Model C N=554	62	36 (58.1)	2.11	1.07-4.17	38	19 (50.0)	1.99	0.86-4.63	83	38 (45.8)	1.42	0.75-2.69	21	11(52.4)	1.41	0.46-4.31	350	127 (36.3)	1.00	--
**Extremely concerned about the COVID-19 pandemic affecting other health outcomes**																				
Model A N=1240	266	244 (91.7)	2.01	1.12-3.61	186	161 (86.6)	1.29	0.71-2.32	232	199 (85.8)	1.38	0.81-2.37	29	22 (75.9)	0.68	0.24-1.89	527	387 (73.4)	1.00	--
Model B N=1240	266	244 (91.7)	1.94	1.08-3.49	186	161 (86.6)	1.25	0.69-2.25	232	199 (85.8)	1.38	0.81-2.37	29	22 (75.9)	0.67	0.24-1.89	527	387 (73.4)	1.00	--
Model C N=554	62	51 (82.3)	1.73	0.78-3.87	38	31 (81.6)	1.64	0.62-4.39	83	54 (65.1)	1.18	0.64-2.15	21	14 (66.7)	0.68	0.22-2.13	350	228 (65.1)	1.00	--
**Extremely concerned about contracting the COVID-19 virus**																				
Model A N=1240	266	252 (94.7)	3.21	1.65-6.27	186	165 (88.7)	1.81	0.97-3.36	232	200 (86.2)	0.88	0.51-1.53	29	24 (82.8)	0.71	0.23-2.20	527	395 (74.9)	1.00	--
Model B N=1240	266	252 (94.7)	2.53	1.30-4.94	186	165 (88.7)	1.41	0.76-2.63	232	200 (86.2)	0.83	0.48-1.45	29	24 (82.8)	0.72	0.23-2.27	527	395 (74.9)	1.00	--
Model C N=554	62	52 (83.9)	1.85	0.84-4.09	38	28 (73.7)	1.52	0.62-3.73	83	54 (65.1)	0.68	0.37-1.26	21	16 (76.2)	0.88	0.27-2.88	350	233 (66.6)	1.00	--
**Extremely concerned about continuing cancer treatment during the COVID-19 pandemic**																				
Model A N=852	186	55 (29.6)	0.80	0.45-1.41	125	36 (28.8)	0.65	0.35-1.23	174	43 (24.7)	0.65	0.35-1.22	20	11 (55.0)	2.44	0.85-7.03	347	90 (25.9)	1.00	--
Model B N=852	186	55 (29.6)	0.84	0.47-1.49	125	36 (28.8)	0.74	0.39-1.40	174	43 (24.7)	0.68	0.36-1.28	20	11 (55.0)	2.38	0.81-6.98	347	90 (25.9)	1.00	--
Model C N=332	40	12 (30.0)	0.50	0.19-1.33	22	9 (40.9)	0.96	0.30-3.03	50	25 (50.0)	2.34	0.99-5.53	14	8 (57.1)	0.90	0.22-3.62	206	69 (33.5)	1.00	--

[a]Model A is regression analysis output with unknown cancer stage values imputed using Multiple Imputation. Model B is sensitivity analysis output with unknown stage values accounted for as a separate category within the cancer stage variable. Model C is complete case analysis output for the 554 participants who provided their cancer stage. Referent group for all comparisons is White. Models were adjusted for apriori selected sociodemographic (gender, age, education, income, insurance status, place of residence) and clinical variables (cancer diagnosis, stage). Abbreviations: OR, odds ratio; CI, confidence interval.
[b] Other race category comprises American Indian/Alaska Native, Native Hawaiian, and Multiple Race.

© 2022 Patel MI et al. *JAMA Network Open.*

eTable 4. Sensitivity Analysis for Social and Economic Impacts by Race and Ethnicity in a National Survey Study of 1240 Adults With Cancer in the United States From September 1, 2020, to January 12, 2021[a]

	African American or Black				Asian				Hispanic or Latinx				Other[b]				White			
	Total Response No.	No. (%)	OR	95% CI	Total Response No.	No. (%)	OR	95% CI	Total Response No.	No. (%)	OR	95% CI	Total Response No.	No. (%)	OR	95% CI	Total Response No.	No. (%)	OR	95% CI
**Experienced job loss**																				
Model A N=1240	266	81 (30.5)	1.61	0.98-2.65	186	6 (3.2)	0.15	0.06-0.37	232	59 (25.4)	1.88	1.04-3.38	29	8 (27.6)	2.96	1.10-7.96	527	86 (16.3)	1.00	--
Model B N=1240	266	81 (30.5)	1.60	0.97-2.64	186	6 (3.2)	0.15	0.06-0.37	232	59 (25.4)	1.86	1.03-3.36	29	8 (27.6)	3.00	1.12-8.07	527	86 (16.3)	1.00	--
Model C N=554	62	15 (24.2)	1.07	0.48-2.36	38	3 (7.9)	0.26	0.07-1.05	83	23 (27.7)	2.44	1.12-5.34	21	7 (33.3)	4.44	1.25-15.75	350	68 (19.4)	1.00	--
**Experienced food insecurity**																				
Model A N=1240	266	230 (86.5)	4.32	2.65-7.04	186	11 (5.9)	1.15	0.72-1.83	232	206 (88.8)	6.13	3.57-10.53	29	19 (65.5)	1.21	0.50-2.93	527	286 (54.3)	1.00	--
Model B N=1240	266	230 (86.5)	4.52	2.77-7.36	186	11 (5.9)	1.27	0.80-2.01	232	206 (88.8)	6.43	3.75-11.02	29	19 (65.5)	1.22	0.50-2.99	527	286 (54.3)	1.00	--
Model C N=554	62	49 (79.0)	3.86	1.82-8.22	38	19 (50.0)	1.03	0.46-2.27	83	60 (72.3)	4.15	2.22-7.77	21	14 (66.7)	2.42	0.84-6.97	350	185 (52.9)	1.00	--
**Experienced childcare loss**																				
Model A N=816	230	91 (39.6)	2.63	1.16-5.97	166	36 (21.7)	2.68	1.12-6.41	188	43 (22.9)	3.22	1.37-7.59	19	7 (36.8)	5.17	1.47-18.11	231	45 (19.5)	1.00	--
Model B N=816	230	91 (39.6)	2.41	1.08-5.36	166	36 (21.7)	2.41	1.04-5.60	188	43 (22.9)	2.98	1.27-6.97	19	7 (36.8)	4.53	1.29-15.84	231	45 (19.5)	1.00	--
Model C N-196	35	14 (40.0)	3.93	0.99-15.58	28	9 (32.1)	2.22	0.42-11.85	43	16 (37.2)	4.60	1.40-15.05	12	6 (50.0)	11.58	1.84-72.92	78	13 (16.7)	1.00	--

[a]Model A is regression analysis output with unknown cancer stage values imputed using Multiple Imputation. Model B is sensitivity analysis output with unknown stage values accounted for as a separate category within the cancer stage variable. Model C is complete case analysis output for the 554 participants who provided their cancer stage. Referent group for all comparisons is White. Models were adjusted for apriori selected sociodemographic (gender, age, education, income, insurance status, place of residence) and clinical variables (cancer diagnosis, stage). Abbreviations: OR, odds ratio; CI, confidence interval.
[b] Other race category comprises American Indian/Alaska Native, Native Hawaiian, and Multiple Race.

© 2022 Patel MI et al. *JAMA Network Open.*

eTable 5. Sensitivity Analysis for Concern Regarding Social and Economic Impacts by Race and Ethnicity in a National Survey Study of 1240 Adults With Cancer in the United States From September 1, 2020, to January 12, 2021[a]

	African American or Black				Asian				Hispanic or Latinx				Other[b]				White			
	Total Response No.	No. (%)	OR	95% CI	Total Response No.	No. (%)	OR	95% CI	Total Response No.	No. (%)	OR	95% CI	Total Response No.	No. (%)	OR	95% CI	Total Response No.	No. (%)	OR	95% CI
**Extremely concerned about the COVID-19 pandemic affecting job security**																				
Model A N=1240	266	237 (89.1)	1.67	0.98-2.85	186	132 (71.0)	0.68	0.41-1.14	232	214 (92.2)	4.71	2.53-8.78	29	26 (89.7)	3.72	1.00-13.82	527	378 (71.7)	1.00	--
Model B N=1240	266	237 (89.1)	1.72	1.01-2.93	186	132 (71.0)	0.74	0.44-1.24	232	214 (92.2)	4.87	2.62-9.06	29	26 (89.7)	3.64	0.98-13.60	527	378 (71.7)	1.00	--
Model C N=554	62	53 (85.5)	2.43	1.05-5.61	38	25 (65.8)	0.88	0.37-2.12	83	66 (79.5)	2.64	1.33-5.25	21	18 (85.7)	3.7	0.94-14.54	350	218 (62.3)	1.00	--
**Extremely concerned about the COVID-19 pandemic affecting food security**																				
Model A N=1240	266	176 (66.2)	2.02	1.34-3.04	186	84 (45.2)	0.99	0.63-1.56	232	168 (72.4)	2.94	1.86-4.66	29	14 (48.3)	1.18	0.51-2.77	527	216 (41.0)	1.00	--
Model B N=1240	266	176 (66.2)	2.06	1.37-3.11	186	84 (45.2)	1.07	0.68-1.69	232	168 (72.4)	3.10	1.95-4.91	29	14 (48.3)	1.19	0.50-2.79	527	216 (41.0)	1.00	--
Model C N=554	62	41 (66.1)	2.47	1.28-4.79	38	14 (36.8)	0.78	0.34-1.80	83	45 (54.2)	2.81	1.53-5.16	21	11 (52.4)	1.58	0.56-4.47	350	124 (35.4)	1.00	--
**Extremely concerned about the COVID-19 pandemic affecting housing security**																				
Model A N=1240	266	253 (95.1)	6.97	3.49-13.92	186	165 (88.7)	3.49	1.86-6.56	232	212 (91.4)	5.75	3.04-10.87	29	22 (75.9)	1.08	0.37-3.18	527	366 (69.4)	1.00	--
Model B N=1240	266	253 (95.1)	6.47	3.22-12.99	186	165 (88.7)	3.18	1.70-5.95	232	212 (91.4)	5.70	3.02-10.75	29	22 (75.9)	1.17	0.39-3.51	527	366 (69.4)	1.00	--
Model C N=554	62	51 (82.3)	3.79	1.73-8.32	38	24 (63.2)	1.40	0.61-3.24	83	64 (77.1)	3.70	1.92-7.13	21	15 (71.4)	1.86	0.61-5.72	350	203 (58.0)	1.00	--
**Extremely concerned about the COVID-19 pandemic affecting childcare[c]**																				
Model A N=816	266	191 (71.8)	2.53	1.12-5.75	186	137 (73.7)	3.60	1.59-8.12	232	124 (53.4)	1.00	0.45-2.18	29	13 (44.8)	1.27	0.35-4.58	527	175 (33.2)	1.00	--
Model B N=816	266	191 (71.8)	2.65	1.18-5.96	186	137 (73.7)	3.83	1.73-8.49	232	124 (53.4)	1.04	0.48-2.25	29	13 (44.8)	1.23	0.34-4.44	527	175 (33.2)	1.00	--
Model C N=196	62	24 (38.7)	2.01	0.47-8.52	38	22 (57.9)	4.23	0.83-21.68	83	29 (34.9)	1.90	0.54-6.71	21	8 (38.1)	1.24	0.17-9.24	350	46 (13.1)	1.00	--
**Extremely concerned about the COVID-19 pandemic affecting financial stability**																				
Model A N=1240	261	251 (96.2)	3.56	1.79-7.08	186	129 (69.4)	0.55	0.31-0.95	232	222 (95.7)	4.29	1.98-9.29	29	28 (96.6)	2.88	0.63-13.14	527	437 (82.9)	1.00	--
Model B N=1240	261	251 (96.2)	3.71	1.86-7.41	186	129 (69.4)	0.60	0.34-1.04	232	222 (95.7)	4.48	2.07-9.71	29	28 (96.6)	2.90	0.64-13.26	527	437 (82.9)	1.00	--
Model C N=554	62	59 (95.2)	6.28	1.75-22.50	38	30 (78.9)	1.25	0.47-3.29	83	76(91 .6)	3.83	1.55-9.46	21	19 (90.5)	2.87	0.58-14.13	350	276 (78.9	1.00	--
**Extremely concerned about the COVID-19 pandemic affecting ability to afford cancer care and treatment**																				

© 2022 Patel MI et al. *JAMA Network Open.*

Model A N=1240	266	251 (94.4)	4.27	2.20-8.28	186	145 (78.0)	0.80	0.46-1.40	232	215 (92.7)	2.81	1.48-5.36	29	26 (89.7)	2.24	0.60-8.34	527	402 (76.3)	1.00	--
Model B N=1240	266	251 (94.4)	4.15	2.14-8.07	186	145 (78.0)	0.83	0.48-1.44	232	215(92.7)	2.92	1.54-5.54	29	26 (89.7)	2.25	0.60-8.36	527	402 (76.3)	1.00	--
Model C N=554	62	50 (80.6)	2.41	1.07-5.42	38	27 (71.1)	0.64	0.26-1.60	83	68(81.9)	2.00	0.97-4.12	21	20 (95.2)	7.89	0.90-68.99	350	240 (68.6)	1.00	--

[a]Model A is regression analysis output with unknown cancer stage values imputed using Multiple Imputation. Model B is sensitivity analysis output with unknown stage values accounted for as a separate category within the cancer stage variable. Model C is complete case analysis output for the 554 participants who provided their cancer stage. Referent group for all comparisons is White. Models were adjusted for apriori selected sociodemographic (gender, age, education, income, insurance status, place of residence) and clinical variables (cancer diagnosis, stage). Abbreviations: OR, odds ratio; CI, confidence interval.

[b] Other race category comprises American Indian/Alaska Native, Native Hawaiian, and Multiple Race.

[c] *In the model A (unknown cancer stage values imputed using multiple imputation) predicting concern about childcare, one iteration of the multiple imputation for unknown cancer stage generated a structural positivity violation in which there were no cases among Stage 0. This imputation was excluded and the results comprise N=99 iterations

© 2022 Patel MI et al. *JAMA Network Open.*

eTable 6. Sensitivity Analysis for Sources of Trust by Race and Ethnicity for COVID-19 Information in a National Survey Study of 1240 Adults With Cancer in the United States From September 1, 2020, to January 12, 2021[a]

	African American or Black				Asian				Hispanic or Latinx				Other[b]				White			
	Total Response No.	No. (%)	OR	95% CI	Total Response No.	No. (%)	OR	95% CI	Total Response No.	No. (%)	OR	95% CI	Total Response No.	No. (%)	OR	95% CI	Total Response No (%)	No. (%)	OR	95% CI
**Trust in the federal government (N=1128)**																				
Model A N=1128	260	20 (7.7)	0.12	0.07-0.21	118	46 (39.0)	0.86	0.51-1.45	223	69 (30.9)	0.36	0.22-0.60	23	4 (17.4)	0.22	0.07-0.73	504	237 (47.0)	1.00	--
Model B N=1128	260	20 (7.7)	0.12	0.07-0.21	118	46 (39.0)	0.98	0.58-1.64	223	69 (30.9)	0.38	0.23-0.63	23	4 (17.4)	0.20	0.06-0.67	504	237 (47.0)	1.00	--
Model C N=512	59	15 (25.4)	.27	0.13-0.55	26	15 (57.7)	1.26	0.50-3.16	78	22 (28.2)	0.31	0.16-0.59	20	4 (20.0)	0.17	0.05-0.58	329	176 (53.5)	1.00	--
**Trust in Doctors (N=1160)**																				
Model A N=1160	260	158 (60.8)	0.55	0.34-0.87	150	129 (86.0)	2.63	1.38-5.01	223	142 (63.7)	0.92	0.54-1.55	23	13 (56.5)	0.49	0.19-1.26	504	396(78.6)	1.00	--
Model B N=1134	260	158 (60.8)	0.57	0.35-0.90	150	129 (86.0)	2.83	1.48-5.39	223	142 (63.7)	0.95	0.56-1.61	23	13 (56.5)	0.45	0.17-1.18	504	396(78.6)	1.00	--
Model C N=494	59	36 (61.0)	0.31	0.15-0.66	34	31 (91.2)	2.95	0.76-11.50	78	66 (84.6)	1.24	0.56-2.79	20	11 (55.0)	0.39	0.12-1.28	329	280 (85.1)	1.00	--
**Trust in community (N=1128)**																				
Model A N=1128	260	206 (53.9)	7.91	4.67-13.41	118	24 (20.3)	2.94	1.55-5.61	223	29 (13.0)	1.94	1.06-3.53	23	9 (39.1)	9.44	3.50-25.44	504	49 (9.7)	1.00	--
Model B N=1128	260	206 (53.9)	7.74	4.57-13.09	118	24 (20.3)	2.80	1.48-5.31	223	29 (13.0)	1.90	1.04-3.47	23	9 (39.1)	9.52	3.51-25.82	504	49 (9.7)	1.00	--
Model C N=512	59	19 (32.2)	4.16	1.91-9.04	26	6 (23.1)	2.21	0.74-6.55	78	22 (28.2)	2.35	1.17-4.73	20	9 (45.0)	7.80	2.41-25.27	329	40 (12.2)	1.00	--
**Trust in church (N=1128)**																				
Model A N=1128	260	129 (49.6)	5.34	3.4-8.38	118	30 (25.4)	1.86	1.05-3.27	223	97 (43.5)	4.88	2.99-7.97	23	11 (47.8)	6.76	2.68-17.03	504	76 (15.1)	1.00	--
Model B N=1128	260	260 (49.6)	5.30	3.37-8.34	118	30 (25.4)	1.80	1.02-3.17	223	97 (43.5)	4.83	2.96-7.89	23	11 (47.8)	7.02	2.77-17.78	504	76 (15.1)	1.00	--
Model C N=512	59	25 (42.4)	4.55	2.24-9.25	26	6 (23.1)	1.80	0.62-5.20	78	35 (44.9)	4.35	2.31-8.17	20	10 (50.0)	5.39	1.81-16.10	329	53 (16.1)	1.00	--

[a]Model A is regression analysis output with unknown cancer stage values imputed using Multiple Imputation. Model B is sensitivity analysis output with unknown stage values accounted for as a separate category within the cancer stage variable. Model C is complete case analysis output for the 554 participants who provided their cancer stage. Referent group for all comparisons is White. Models were adjusted for apriori selected sociodemographic (gender, age, education, income, insurance status, place of residence) and clinical variables (cancer diagnosis, stage). Abbreviations: OR, odds ratio; CI, confidence interval.

[b] Other race category comprises American Indian/Alaska Native, Native Hawaiian, and Multiple Race

© 2022 Patel MI et al. *JAMA Network Open.*

eAppendix. Survey Invitation and Survey

Dear Participant,

I invite you to participate in a research study entitled: *Impact of the Coronavirus (COVID-19) on patients with cancer*. This study is being conducted by Dr. Manali Patel from Stanford University.

The purpose of this study is to enhance our knowledge of the evolving COVID-19 pandemic among patients with cancer. The data we plan to collect will help us track the spread of the disease among patients with cancer in our community and to better understand how your cancer care is being affected as well as the actions you are taking to prevent or reduce the impact of COVID-19.

The study involves filling an online survey that should take approximately 5-10 minutes to complete. This survey will ask about current health status, your household, and the actions you have taken thus far in response to the outbreak. We will also ask for your email address if you are willing to complete future surveys to help track the continued impact of the disease.

We believe there are no known risks associated with this research study; however, as with any online related activity the risk of a breach is always possible. To the best of our ability your answers in this study will remain confidential. We will minimize any risks by all information remaining anonymous. The online survey will be numerically coded and kept in a password protected website with access only by the Principal Investigator.

Your participation in this study is completely voluntary and you can withdraw at any time. You are free to skip any question on the online survey that you are not comfortable answering.
Attached is the consent form, which includes specific detailed information regarding the study and the risks and benefits that will be associated to your participation throughout this study.
If you agree to participate in this project, please click the link below to direct you to the survey.
Please answer the questions as best you can.

If you want to participate, click this link to start the survey:

Follow this link to the Survey:
https://stanforduniversity.qualtrics.com/jfe/form/SV_ctKS6ZSMYdEYoXX

If you require additional information or need assistance filing out this survey, **please contact me at**:
Protocol Director: Dr. Manali I. Patel
Email: manalip@stanford.edu
Phone: 650-736-2768
Office Location: 1701 Page Mill Rd. Palo Alto, CA 94304 RM#107

This research has been reviewed according to Stanford University IRB procedures for research involving human subjects. If you have any concerns, complaints, or general questions about your rights as a participant, please contact the Stanford Institutional Review Board (IRB) to speak to someone independent of the research team at 1-866-680-2906.

Sincerely yours,
Manali I. Patel, MD MPH MPS

Impact of the Coronavirus (COVID-19) on patients with cancer

Q1 Have you ever been diagnosed with cancer?

- ○ Yes
- ○ No
- ○ I do not know

Q2 How concerned are you about the novel Coronavirus (COVID-19) pandemic affecting your cancer care?

- ○ Not at all concerned
- ○ A little concerned
- ○ Moderately concerned
- ○ Very concerned
- ○ Extremely concerned

Q3 Have you avoided going to your doctor’s office, urgent care, emergency department or hospital because of fear of getting the novel Coronavirus (COVID-19)?

- ○ Yes
- ○ No

Q4 Are you currently receiving any of the following treatments for your most recent cancer? (Check all that apply)

- ☐ A treatment for my cancer that I receive in my vein (chemotherapy and/or immunotherapy)
- ☐ A treatment for my cancer that I take by mouth (oral chemotherapy or hormonal therapy)
- ☐ Radiation Therapy
- ☐ Surgery
- ☐ I am currently not being treated for my cancer
- ☐ My cancer is in remission
- ☐ I don’t know
- ☐ Other: ____

Branching Logic-Display Question 5:

If Are you currently receiving any of the following treatments for your most recent cancer? = A treatment for my cancer that I receive in my vein (chemotherapy and/or immunotherapy)

Q5 How often do you receive this treatment by vein?

- ○ Once
- ○ Everyday
- ○ Every week
- ○ Every two weeks
- ○ Every three weeks
- ○ Every month
- ○ Other: ____

Branching Logic-Display Question 6:

If Are you currently receiving any of the following treatments for your most recent cancer? = A treatment for my cancer that I take by mouth (oral chemotherapy or hormonal therapy)

Q6 How often do you receive this treatment that you take by mouth?

- ○ Once
- ○ Everyday
- ○ Every week

© 2022 Patel MI et al. *JAMA Network Open.*

- ○ Every two weeks
- ○ Every three weeks
- ○ Every month
- ○ Other: ____

Branching Logic-Display Question 7:

If Are you currently receiving any of the following treatments for your most recent cancer? = Radiation Therapy

Q7 How often do you receive radiation?

- ○ I have only received radiation once
- ○ I receive radiation five times a week
- ○ Other ____

Branching Logic-Display Question 8:

If Are you currently receiving any of the following treatments for your most recent cancer? = A treatment for my cancer that I receive in my vein (chemotherapy and/or immunotherapy)

Or Are you currently receiving any of the following treatments for your most recent cancer? = A treatment for my cancer that I take by mouth (oral chemotherapy or hormonal therapy)

Or Are you currently receiving any of the following treatments for your most recent cancer? = Radiation Therapy

Q8 What are your concerns about receiving your cancer treatment during the novel Coronavirus (COVID-19) pandemic?

- ○ I feel more at risk of getting the Coronavirus (COVID-19) virus if I continue my cancer treatments during the novel Coronavirus (COVID-19) pandemic
- ○ I am not concerned about receiving cancer treatments during the novel Coronavirus (COVID-19) pandemic
- ○ Other: ____

Q9 Are you participating in a cancer clinical trial?

- ○ Yes
- ○ No
- ○ I was planning to enroll in a trial but it was delayed due to the novel Coronavirus (COVID-19) pandemic
- ○ I do not know

<u>Care Delays or Interruptions</u>

Q10 Have you experienced any of the following due to the novel Coronavirus (COVID-19) pandemic? (Check all that apply)

- ☐ My cancer treatment or cancer care (clinic, chemotherapy, radiation, surgery, blood tests, imaging (CT scan, MRI, chest x-ray), clinical trial, or other care) was delayed or interrupted
- ☐ My cancer treatment was switched to another cancer treatment (for example taking a treatment by mouth instead of receiving it by IV in my vein or switching treatments that I receive by IV in my vein?
- ☐ The location of my cancer care was switched (for example receiving cancer care closer to my home or in my home instead of in the clinic or hospital)?
- ☐ I am now taking a new growth factor support drug to protect myself from infection (for example neupogen, onpro, neulasta)
- ☐ I am now taking an antibiotic to protect myself from infection (for example clindamycin, penicillin)
- ☐ I am now taking a new drug to protect myself from blood clots (for example lovenox, enoxaparin,

© 2022 Patel MI et al. *JAMA Network Open.*

coumadin, heparin, argatroban)
- ☐ I have experienced no changes in my care due to the novel Coronavirus (COVID-19) pandemic
- ☐ Other ____

Branching Logic-Display Question 11:

If Have you experienced any of the following due to the novel Coronavirus (COVID-19) pandemic? = My cancer treatment was switched to another cancer treatment (for example taking a treatment by mouth instead of receiving it by IV in my vein or switching treatments that I receive by IV in my vein?

Or Have you experienced any of the following due to the novel Coronavirus (COVID-19) pandemic? = The location of my cancer care was switched (for example receiving cancer care closer to my home or in my home instead of in the clinic or hospital)?

Or Have you experienced any of the following due to the novel Coronavirus (COVID-19) pandemic? = My cancer treatment or cancer care (clinic, chemotherapy, radiation, surgery, blood tests, imaging (CT scan, MRI, chest x-ray), clinical trial, or other care) was delayed or interrupted

Or Have you experienced any of the following due to the novel Coronavirus (COVID-19) pandemic? = Other Is Not Empty

Or Have you experienced any of the following due to the novel Coronavirus (COVID-19) pandemic? (= Other

Q11 Why did you experience this change in your cancer care?

- ○ My doctor requested it due to the novel Coronavirus (COVID-19) pandemic
- ○ I requested it due to the novel Coronavirus (COVID-19) pandemic
- ○ I do not know why I experienced this change in my care
- ○ Other ____

Branching Logic-Display Question 12:

If Have you experienced any of the following due to the novel Coronavirus (COVID-19) pandemic? = My cancer treatment or cancer care (clinic, chemotherapy, radiation, surgery, blood tests, imaging (CT scan, MRI, chest x-ray), clinical trial, or other care) was delayed or interrupted

Q12 What specific cancer care has been delayed due to the novel Coronavirus (COVID-19) pandemic (Check all that apply).

- ☐ Routine clinic visit
- ☐ Cancer treatment that requires an infusion given with an IV (Chemotherapy or immunotherapy)
- ☐ Cancer treatment that I take by mouth (chemotherapy or hormonal therapy)
- ☐ Clinical trial enrollment or other related clinical trial activities
- ☐ Radiation
- ☐ Surgery or biopsy
- ☐ Lab testing (blood work)
- ☐ Imaging (for example, CT scans, x-rays, mammogram, MRI)
- ☐ I do not know
- ☐ Other: ____

Branching Logic-Display Question 13:

If What specific cancer care has been delayed due to the novel Coronavirus (COVID-19) pandemic = Cancer treatment that requires an infusion given with an IV (Chemotherapy or immunotherapy)

Or What specific cancer care has been delayed due to the novel Coronavirus (COVID-19) pandemic = Cancer treatment that I take by mouth (chemotherapy or hormonal therapy)

Q13 How much of a delay or interruption have you had in your cancer treatment that you take by vein or by mouth due to the novel Coronavirus (COVID-19) pandemic?

© 2022 Patel MI et al. *JAMA Network Open.*

- ○ No delay
- ○ Less than 1 week
- ○ 1 week
- ○ 2 weeks
- ○ 3 weeks
- ○ 4 weeks
- ○ More than 4 weeks
- ○ My treatment has been interrupted indefinitely
- ○ I don't know
- ○ Other: ____

Branching Logic-Display Question 14:

If What specific cancer care has been delayed due to the novel Coronavirus (COVID-19) pandemic = Routine clinic visit

Q14 How much of a delay or interruption have you had in your routine clinic visit due to the novel Coronavirus (COVID-19) pandemic?

- ○ No delay
- ○ Less than 1 week
- ○ 1 week
- ○ 2 weeks
- ○ 3 weeks
- ○ 4 weeks
- ○ More than 4 weeks
- ○ Indefinitely
- ○ I do not know
- ○ Other: ____

Branching Logic-Display Question 15:

If What specific cancer care has been delayed due to the novel Coronavirus (COVID-19) pandemic = Radiation

Q15 How much of a delay or interruption have you had in your Radiation due to the novel Coronavirus (COVID-19) pandemic?

- ○ No delay
- ○ Less than 1 week
- ○ 1 week
- ○ 2 weeks
- ○ 3 weeks
- ○ 4 weeks
- ○ More than 4 weeks
- ○ My treatment has been interrupted indefinitely
- ○ I do not know
- ○ Other: ____

Branching Logic-Display Question 16:

If What specific cancer care has been delayed due to the novel Coronavirus (COVID-19) pandemic = Surgery or biopsy

Q16 How much of a delay or interruption have you had in your surgery or biopsy due to the novel Coronavirus (COVID-19) pandemic?

- ○ No delay
- ○ Less than 1 week
- ○ 1 week

© 2022 Patel MI et al. *JAMA Network Open.*

- 2 weeks
- 3 weeks
- 4 weeks
- More than 4 weeks
- Indefinitely
- I do not know
- Other: ____

Branching Logic-Display Question 17:

If What specific cancer care has been delayed due to the novel Coronavirus (COVID-19) pandemic = Lab testing (blood work)

Q17 How much of a delay or interruption have you had in your lab testing (blood work) due to the novel Coronavirus (COVID-19) pandemic?

- No delay
- Less than 1 week
- 1 week
- 2 weeks
- 3 weeks
- 4 weeks
- More than 4 weeks
- Indefinitely
- I do not know
- Other: ____

Branching Logic-Display Question 18:

If What specific cancer care has been delayed due to the novel Coronavirus (COVID-19) pandemic = Imaging (for example, CT scans, x-rays, mammogram, MRI)

Q18 How much of a delay or interruption have you had in your imaging exams (CT scans, MRI, chest x-ray, bone scan, etc) due to the novel Coronavirus (COVID-19) pandemic?

- No delay
- Less than 1 week
- 1 week
- 2 weeks
- 3 weeks
- 4 weeks
- More than 4 weeks
- Indefinitely
- I do not know
- Other: ____

Branching Logic-Display Question 19:

If What specific cancer care has been delayed due to the novel Coronavirus (COVID-19) pandemic = Clinical trial enrollment or other related clinical trial activities

Q19 How much of a delay or interruption have you had in a clinical trial due to the novel Coronavirus (COVID-19) pandemic?

- No delay
- Less than 1 week
- 1 week
- 2 weeks
- 3 weeks
- 4 weeks
- More than 4 weeks

© 2022 Patel MI et al. *JAMA Network Open.*

- ○ Indefinitely
- ○ I do not know
- ○ Other: ____

Branching Logic-Display Question 20:

If Have you experienced any of the following due to the novel Coronavirus (COVID-19) pandemic? = My cancer treatment or cancer care (clinic, chemotherapy, radiation, surgery, blood tests, imaging (CT scan, MRI, chest x-ray), clinical trial, or other care) was delayed or interrupted

Or Have you experienced any of the following due to the novel Coronavirus (COVID-19) pandemic? = My cancer treatment was switched to another cancer treatment (for example taking a treatment by mouth instead of receiving it by IV in my vein or switching treatments that I receive by IV in my vein?

Or Have you experienced any of the following due to the novel Coronavirus (COVID-19) pandemic? = The location of my cancer care was switched (for example receiving cancer care closer to my home or in my home instead of in the clinic or hospital)?

Or Have you experienced any of the following due to the novel Coronavirus (COVID-19) pandemic? = I am now taking a new growth factor support drug to protect myself from infection (for example neupogen, onpro, neulasta)

Or Have you experienced any of the following due to the novel Coronavirus (COVID-19) pandemic? = Other

Or Have you experienced any of the following due to the novel Coronavirus (COVID-19) pandemic? = Other Is Not Empty

Q20 Are you worried that this change in your cancer care due to the novel Coronavirus (COVID-19) could affect your cancer?

- ○ Yes
- ○ No
- ○ I do not know

Branching Logic-Display Question 21:

If Have you experienced any of the following due to the novel Coronavirus (COVID-19) pandemic? = My cancer treatment or cancer care (clinic, chemotherapy, radiation, surgery, blood tests, imaging (CT scan, MRI, chest x-ray), clinical trial, or other care) was delayed or interrupted

Q21 Which of the following occurred when your cancer care was delayed or interrupted?

- ▢ My cancer doctor called me by phone at my scheduled appointment time
- ▢ My cancer doctor conducted a video visit with me
- ▢ My appointment, lab and/or imaging tests were rescheduled to a later date
- ▢ My appointment was cancelled indefinitely
- ▢ None of these occurred
- ▢ Other: ____

Branching Logic-Display Question 22:

If Which of the following occurred when your cancer care was delayed or interrupted? = My cancer doctor called me by phone at my scheduled appointment time

Q22 On a scale of 0-10 with 0 being not satisfied at all and 10 being the most satisfied, how satisfied were you with your phone call?

- ○ 0
- ○ 1
- ○ 2
- ○ 3

© 2022 Patel MI et al. *JAMA Network Open.*

- 4
- 5
- 6
- 7
- 8
- 9
- 10

Branching Logic-Display Question 23:

If Which of the following occurred when your cancer care was delayed or interrupted? = My cancer doctor conducted a video visit with me

Q23 On a scale of 0-10 with 0 being not satisfied at all and 10 being the most satisfied, how satisfied were you with your video visit?

- 0
- 1
- 2
- 3
- 4
- 5
- 6
- 7
- 8
- 9
- 10

Branching Logic-Display Question 24:

If Which of the following occurred when your cancer care was delayed or interrupted? = My cancer doctor called me by phone at my scheduled appointment time

Or Which of the following occurred when your cancer care was delayed or interrupted? = My cancer doctor conducted a video visit with me

Q24 Could you tell us more about your experience with the phone call or video visit? (For example, what could be improved?)

type your response: ____

Q25 In the future, if your scheduled routine cancer care appointment was delayed due to the novel Coronavirus (COVID-19) pandemic, which of the following would you choose? (Please select one)

- Wait until my cancer doctor is available and reschedule an in-person visit.
- Reschedule an in-person visit with another cancer doctor on the same day
- Talk to my cancer doctor by phone at the time of my original appointment
- Set up a video visit at the time of my original appointment
- Cancel my appointment until when safe to return to clinic

Q26 It is always important to discuss your health care wishes with your family and doctor and to have these wishes in writing in case you are too sick to make decisions for yourself.
Have you discussed your health care wishes with your doctor in the past?

- Yes
- No
- I do not know

© 2022 Patel MI et al. *JAMA Network Open.*

Branching Logic-Display Question 27:

If It is always important to discuss your health care wishes with your family and doctor and to have these wishes in writing in case you are too sick to make decisions for yourself. Have you discussed your health care wishes with your doctor in the past? = Yes

Q27 Has the novel Coronavirus (COVID-19) pandemic changed your views or your wishes about health care if you could not breathe?

- ○ Yes
- ○ No
- ○ I do not know

Branching Logic-Display Question 28:

If It is always important to discuss your health care wishes with your family and doctor and to have these wishes in writing in case you are too sick to make decisions for yourself. Have you discussed your health care wishes with your doctor in the past? = Yes

And If It is always important to discuss your health care wishes with your family and doctor and to have these wishes in writing in case you are too sick to make decisions for yourself. Have you discussed your health care wishes with your doctor in the past? = I do not know

Q28 Would you like to discuss your health care wishes again with your doctors in the setting of the novel Coronavirus (COVID-19) pandemic?

- ○ Yes
- ○ No
- ○ I do not know

Branching Logic-Display Question 29:

If It is always important to discuss your health care wishes with your family and doctor and to have these wishes in writing in case you are too sick to make decisions for yourself. Have you discussed your health care wishes with your doctor in the past? = No

Q29 Please explain what barriers may have contributed to this discussion with your doctor?

- ○ The discussion was not brought up by my doctors
- ○ I did not wish to discuss my health care wishes with my doctors at the time
- ○ I do not know why we did not discuss this together
- ○ Other: ____

Challenges due to the novel Coronavirus (COVID-19) Pandemic

Q30 Have you experienced any difficulties due to the novel Coronavirus (COVID-19)? (Check all that apply)

- ▢ I have experienced reduced job wages or work hours
- ▢ I have experienced job loss
- ▢ I have experienced difficulties getting food
- ▢ I have experienced difficulties getting childcare
- ▢ I have experienced difficulties getting transportation
- ▢ I have experienced difficulties accessing cancer care or other health care services
- ▢ I have experienced difficulties getting routine or essential medications
- ▢ Other: ____

Q31 How concerned do you feel about the Coronavirus (COVID-19) pandemic? (Please select one)

- ○ Not at all concerned
- ○ A little concerned
- ○ Moderately concerned

© 2022 Patel MI et al. *JAMA Network Open.*

- ○ Very concerned
- ○ Extremely concerned

Q32 Please rank how concerned you are about the following due to the novel Coronavirus (COVID-19) pandemic on a scale of 0-10 (0 being not concerned at all and 10 being extremely concerned).

	Not Concerned 0 1 2 3 4 5 6 7 8 9 10 Extremely Concerned
I will not be able to get food	
I will not be able to work	
I will lose housing	
I will not have childcare	
I will not be able to pay for cancer treatment or healthcare	
I will have other financial concerns	
My cancer will worsen	
My other health conditions will worsen	
I will get the virus	
Someone in my family will get the virus	
Other:	

Q33 How often do you feel overwhelmed by concerns related to the novel Coronavirus (COVID-19)?

- ○ Never
- ○ Rarely
- ○ Sometimes
- ○ Often
- ○ Always

Q34 Have you spoken with your cancer doctor about any of these concerns due to the novel Coronavirus (COVID-19)?

- ○ Yes
- ○ No

Branching Logic-Display Question 35:

If Have you spoken with your cancer doctor about any of these concerns due to the novel Coronavirus (COVID-19)? = No

Q35 Please explain what barriers may have contributed to discussing these concerns with your doctor?

© 2022 Patel MI et al. *JAMA Network Open.*

(Check all that apply)

- ▢ I could not reach my cancer doctor
- ▢ I did not want to bother my cancer doctor
- ▢ I did not think about discussing these concerns with my cancer doctor
- ▢ I discussed these concerns with other health care professionals (such as my primary care, nurse, social worker)
- ▢ I do not know
- ▢ Other

Q36 What sources do you trust regarding the novel Coronavirus (COVID-19) pandemic? (Check all that apply)

- ▢ Government (local or federal)
- ▢ News (for example newspaper, TV)
- ▢ Friends or family members
- ▢ Church/pastor
- ▢ Local community agencies
- ▢ Social media (for example Twitter, Facebook, Instagram)
- ▢ Doctors
- ▢ Cancer advocacy or support groups (for example American Cancer Society Cancer Action Network, Susan G Komen, American Society of Clinical Oncology)
- ▢ Other: ____

Q37 What information from your cancer doctor would be most useful about the novel Coronavirus (COVID-19)? (Check all that apply)

- ▢ I feel well informed about the novel Coronavirus (COVID-19)
- ▢ I would like my doctor to provide me with information about Coronavirus (COVID19) symptoms to look out for
- ▢ I would like my doctor to provide me with information about how to prevent exposure to the novel Coronavirus (COVID-19)
- ▢ I would like my doctor to provide me with information about my risk of getting the novel Coronavirus (COVID-19)
- ▢ Other: ____

Q38 How interested would you be in participating in a free online mindfulness-based stress reduction training program?

- ○ Not at all interested
- ○ Somewhat interested
- ○ Very interested
- ○ Extremely interested

<u>Novel Coronavirus (COVID-19) Testing</u>

Q39 Have you been tested for the novel Coronavirus (COVID-19)?

- ○ Yes, I was tested, and the test was positive for the novel Coronavirus (COVID-19)
- ○ Yes, I was tested, and the test was negative for the novel Coronavirus (COVID-19)
- ○ No, I tried to get tested but could not get a test for the novel Coronavirus (COVID-19)
- ○ No, I have not tried to get tested for the novel Coronavirus (COVID-19)

© 2022 Patel MI et al. *JAMA Network Open.*

Branching Logic-Display Question 40:

If Have you been tested for the novel Coronavirus (COVID-19)? = Yes, I was tested, and the test was positive for the novel Coronavirus (COVID-19)

Or Have you been tested for the novel Coronavirus (COVID-19)? = No, I tried to get tested but could not get a test for the novel Coronavirus (COVID-19)

Q40 Did you have to go to the Emergency Room for symptoms due to the novel Coronavirus (COVID-19)?

- ○ Yes
- ○ No
- ○ I do not know

Branching Logic-Display Question 41:

If Have you been tested for the novel Coronavirus (COVID-19)? = Yes, I was tested, and the test was positive for the novel Coronavirus (COVID-19)

Or Have you been tested for the novel Coronavirus (COVID-19)? = No, I tried to get tested but could not get a test for the novel Coronavirus (COVID-19)

Q41 Did you have to go to the be hospitalized for symptoms due to the novel Coronavirus (COVID-19)?

- ○ Yes
- ○ No
- ○ I do not know

Branching Logic-Display Question 42:

If Have you been tested for the novel Coronavirus (COVID-19)? = Yes, I was tested, and the test was positive for the novel Coronavirus (COVID-19)

Or Have you been tested for the novel Coronavirus (COVID-19)? = No, I tried to get tested but could not get a test for the novel Coronavirus (COVID-19)

Q42 Did you have to stay in an intensive care unit for symptoms due to the novel Coronavirus (COVID-19)?

- ○ Yes
- ○ No
- ○ I do not know

Q43 Has anyone in your household been tested for the novel Coronavirus (COVID-19)?

- ○ Yes, they were tested, and the test was positive for the novel Coronavirus (COVID-19)
- ○ Yes, they were tested, and the test was negative for the novel Coronavirus (COVID-19)
- ○ No, they tried to get tested, but could not get a test for the novel Coronavirus (COVID-19)
- ○ No, they did not try to get tested for the novel Coronavirus (COVID-19)

<u>Demographic Information</u>

Q44 What month and year were you diagnosed with cancer? (Select Month and Year from dropdown)

Q45 What was the diagnosis of your most recent cancer?

- ○ Bladder Cancer
- ○ Breast Cancer
- ○ Colon Cancer
- ○ Gallbladder Cancer
- ○ Rectal Cancer
- ○ Esophageal Cancer
- ○ Stomach Cancer
- ○ Liver Cancer
- ○ Brain Cancer
- ○ Kidney Cancer

- Lung Cancer
- Lymphoma
- Melanoma
- Head and neck cancer
- Pancreas Cancer
- Prostate Cancer
- Testicular Cancer
- Thyroid Cancer
- Uterine Cancer
- Ovarian Cancer
- Cervical Cancer
- Leukemia
- Multiple Myeloma
- Myelodysplastic syndrome
- Sarcoma
- Cancer of unknown origin
- I don't know
- Other:

Q46 What was the stage of your cancer at diagnosis?

- Stage 0 (in situ cancer)
- Stage 1
- Stage 2
- Stage 3
- Stage 4
- I don't know

Q47 Is your most recent cancer considered curable?

- Yes
- No
- I don't know

Q48 Please enter the zip code of your residence.

Zip Code ____

Q49 What is the highest level of school you have completed or the highest degree you have received?

- Less than high school degree
- High school graduate (high school diploma or equivalent including GED)
- Some college but no degree
- Associate degree in college (2-year)
- Bachelor's degree in college (4-year)
- Master's degree
- Doctoral degree
- Professional degree (JD, MD)

Q50 What is your employment status?

- Employed full time
- Employed part time
- Self-employed
- Unemployed looking for work
- Unemployed not looking for work
- Retired
- Student
- Disabled

© 2022 Patel MI et al. *JAMA Network Open.*

Branching Logic-Display Question 51:

If What is your employment status? = Unemployed looking for work

Or What is your employment status? = Unemployed not looking for work

Q51 Are you unemployed due to the novel Coronavirus (COVID-19) pandemic?

- ○ Yes
- ○ No
- ○ I don't know
- ○ Other: ____

Branching Logic-Display Question 52:

If Are you unemployed due to the novel Coronavirus (COVID-19) pandemic? = Yes

Q52 Have you applied for disability?

- ○ Yes
- ○ No
- ○ I don't know

Branching Logic-Display Question 53:

If Have you applied for disability? = Yes

Q53 Have you had any delays getting disability due to the novel Coronavirus (COVID-19) pandemic?

- ○ Yes
- ○ No
- ○ I don't know

Q54 Do you have access to any of the following at home? (Check all that apply)

- ☐ Smartphone
- ☐ Desktop or laptop computer
- ☐ Tablet
- ☐ Internet access at home
- ☐ Home telephone (landline)

Q55 Do you have health insurance?

- ○ Yes
- ○ No
- ○ I no longer have health insurance due to the novel Coronavirus (COVID-19) pandemic
- ○ I do not know

Branching Logic-Display Question 56:

If Do you have health insurance? = Yes

Q56 What is your health insurance? (Check all that apply)

- ☐ Private insurance that I get from my job
- ☐ Private insurance that is not provided through my job
- ☐ Public insurance that I get from the county or the government such as Medicaid
- ☐ Medicare
- ☐ Uninsured
- ☐ Cash
- ☐ I do not know

© 2022 Patel MI et al. *JAMA Network Open.*

- ☐ Other ____

Q57 What gender do you currently identify as?

- ○ Male
- ○ Female
- ○ Non-binary
- ○ Prefer to not answer
- ○ Other

Q58 What is your annual household income?

- ○ Less than $25,000
- ○ $25,000 to $34,999
- ○ $35,000 to $49,999
- ○ $50,000 to $74,999
- ○ $75,000 to $99,999
- ○ $100,000 to $149,999
- ○ $150,000 or more
- ○ Prefer to not answer

Q59 What year were you born?

Dropdown list (1920-2020)

Q60 What is your marital status?

- ○ Married
- ○ Divorced
- ○ Widowed
- ○ Separated
- ○ Never Married
- ○ A member of an unmarried couple

Q61 How many people are living in your household (other than yourself)?

- ○ I live alone
- ○ 1
- ○ 2
- ○ 3
- ○ 4
- ○ 5
- ○ 6
- ○ > 6

Q62 How many adults age 65 years or older live in your household (other than yourself if you are 65 years or older)?

- ○ 0
- ○ 1
- ○ 2
- ○ 3
- ○ 4
- ○ 5
- ○ 6
- ○ > 6

Q63 How many people age 18 years or younger live in your household?

- ○ 0
- ○ 1
- ○ 2

© 2022 Patel MI et al. *JAMA Network Open.*

- 3
- 4
- 5
- 6
- > 6

Q64 Which of the following would you say is your race?

- ☐ White
- ☐ Black or African American
- ☐ American Indian or Alaska Native
- ☐ Asian
- ☐ Native Hawaiian or Pacific Islander
- ☐ Prefer to not answer
- ☐ Race not listed above: ____

Q65 Are you Spanish, Hispanic, or Latino?

- Yes
- No

Q66 What is the primary language you speak at home?

- English
- Spanish
- Other: ____

Branching Logic-Display Question 67:

If What is the primary language you speak at home? = Spanish

Or What is the primary language you speak at home? = Other

Q67 Since you speak another language other than English at home, we are interested in your own opinion of how well you speak English. Would you say you speak English:

- Very well
- Well
- Not Well
- Not at all

Q68 If you are willing to complete a future survey or interview, please enter your email address and/or your phone number.

Email Address: ____

Phone Number: ____

Important Note: If you are concerned that you or a family member may be infected with COVID-19 (novel coronavirus), please contact your primary care physician or local healthcare provider.

© 2022 Patel MI et al. *JAMA Network Open.*
